# Integrated multi-omics profiling of treatment-naive cervix uteri premalignant lesions and cervical squamous cell carcinoma reveals ecosystem and drivers underlying cervical cancer progression

**DOI:** 10.1038/s41419-026-08960-2

**Published:** 2026-06-09

**Authors:** Yubing Zhou, Yanlin Luo, Yunxiao Ge, Xinxin Wu, Rui Wu, Hangrui Liu, Lin Tang, Yuanying Li, Chenchen Ren, Xianxu Zeng, Yamei Hu, Kangdong Liu, Hui Liu, Zigang Dong

**Affiliations:** 1 Department of Pathophysiology, School of Basic Medical Sciences, College of Medicine, Zhengzhou University, Zhengzhou, Henan China; 2 China-US (Henan) Hormel Cancer Institute, Zhengzhou, Henan China; 3 The Affiliated Cancer Hospital of Zhengzhou University, Henan Cancer Hospital, Zhengzhou, Henan China; 4 Department of Pathology, The Third Affiliated Hospital of Zhengzhou University, Zhengzhou University, Zhengzhou, Henan China; 5 Department of Physiology and Pharmacology, Karolinska Institutet, Stockholm, Sweden

**Keywords:** Tumour biomarkers, Oncogenes, Cancer prevention, Cancer microenvironment

## Abstract

The tumorigenesis and progression of conventional cervical squamous cell carcinoma generally follow a well-defined cascade: from normal cervix uteri to high-grade cervical intraepithelial neoplasia (precancer), cervical carcinoma in situ, and ultimately invasive cervical carcinoma. However, biomarkers and targets that reflect tumor evolution during CSCC progression at different time points within the same patient remain lacking. We integrated single-cell RNA sequencing with spatial transcriptomics from a series of human cervix uteri tissues spanning the cascade of cervical premalignant lesions and malignant progression obtained from the same patients. We found that fibroblasts and endothelial cells decreased, whereas immune cells (mainly T cells and B cells) increased during CSCC development. Moreover, the immune response-related GNLY-fibroblasts and GNLY-endothelial cells were significantly enriched during the PCT of CSCC. Additionally, a panel of gene signatures (CLDN1, ISG15, PTGDS) with clinical significance for the precise diagnosis and personalized treatment strategies of PCT was identified. This gene panel may serve as an auxiliary diagnostic indicator in cervical biopsy specimens and help identify lesions associated with malignant progression. CLDN1 and ISG15 promote CSCC progression, whereas PTGDS suppresses it. Moreover, ISG15, synthesized by inflammatory cancer-associated fibroblasts (CAFs), enhances the stability of FGF1, thereby activating the FGF1/FGFR1/PI3K/AKT/mTOR signaling pathway. FGFR1 and PI3K/AKT/mTOR inhibitors were found to suppress CSCC cell proliferation in vitro and tumor growth in vivo. High ISG15 and CLDN1 expression correlated with poor survival in cervical cancer tissue microarrays and with reduced immunotherapy response in exploratory public datasets, pending validation in CSCC cohorts. Our study defines the ecosystem of PCT from cell subpopulations, the communicating gene networks, and key signal transductions involved in PCT of CSCC. These findings offer new insights into the spatiotemporal evolution of the human cervix in premalignant and malignant lesions, paving the way for improved CSCC diagnosis and therapy.

**Figure:**
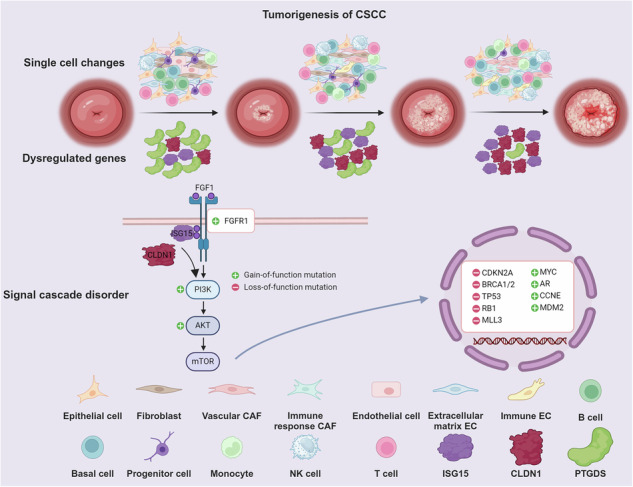

## Highlights

- A single-cell and spatial transcriptomic atlas of premalignant lesions and cervical squamous cell carcinoma (CSCC) derived from the same patients was constructed.
- The immune response-related GNLY-fibroblasts and GNLY-endothelial cells were significantly enriched during precancer-to-cancer transition (PCT) of CSCC.
- A gene panel comprising CLDN1, ISG15, and PTGDS was identified as potential biomarkers for PCT. Elevated CLDN1 and ISG15 expression are associated with poor overall survival in cervical cancer.
- CLDN1 and ISG15 promote CSCC progression, while PTGDS suppresses it. Mechanistically, ISG15 stabilizes FGF1 and activates the FGF1/FGFR1/PI3K/AKT/mTOR signaling pathway. Inhibitors targeting this pathway may have therapeutic potential in cervical cancer.

## Introduction

In 2022, there were approximately 662,301 new cervix uteri cancer cases and 348,874 related deaths worldwide. Cervical cancer remains a major cause of cancer incidence and mortality among women worldwide. The etiology of cervical cancer has been extensively investigated. Human papillomavirus (HPV) infection is recognized as the leading risk factor, whereas human immunodeficiency virus (HIV) infection, smoking, and unsafe sexual behavior are contributing risk factors for cervical cancer [1]. Treatment is determined by the disease stage at diagnosis. However, the overall prognosis for women with metastatic or recurrent disease remains poor due to inoperability [2, 3]. Thus, it is essential to elucidate the spatiotemporal transformation ecosystem of the cervix uteri in premalignant and malignant lesions, with the goal of intercepting cervical cancer at its earliest stages. Cervical cancer can be classified into cervical squamous cell carcinoma, cervical adenocarcinoma, cervical adenosquamous carcinoma, and neuroendocrine carcinoma according to histopathology. CSCC accounts for approximately 80%, whereas cervical adenocarcinoma accounts for about 10–20% of all cervical cancer types [4]. The changes in cell types, spatial distribution, and gene networks play critical roles in the cascade of PCT. However, the full spectrum of diverse cell types, spatiotemporal transformation, and gene networks remains to be explored in the cervix uteri of premalignant and malignant lesions, especially from the same patients, which limits our ability to investigate their contributions to cervical cancer pathogenesis. Cellular characteristics play a crucial role in the identification of premalignant and malignant lesions in the cervix. In the healthy cervix uteri, the epithelium consists of a variety of cell types, including epithelial cells, smooth muscle cells, fibroblasts, endothelial cells, endometrial stromal cells, and immune cells. These cell types function together to maintain morphogenesis and preserve tissue homeostasis [5]. The damage caused by the HPV infection and other risk factors to the cervix uteri is characterized by the partial and, eventually, total occupation of squamous epithelial cells, thus developing as CSCC. However, the exact pathogenesis of CSCC remains poorly understood, especially during the premalignant-to-malignant transition. Previous studies on molecular characteristics of intra-tumoral heterogeneity, chemotherapy resistance, and radioresistance in cervical cancer have failed to fully distinguish among cell types, capture the spatiotemporal transformation, or validate biomarkers and therapeutic targets of CSCC with specific states [5–7]. Consequently, a comprehensive and systematic profile of the cell types, spatiotemporal transformation, and gene signatures in cervix uteri premalignant and malignant tissue profiles is urgently needed.

The advent of single-cell RNA sequencing (scRNA-seq) and spatial transcriptomics (ST) has dramatically enhanced our ability to thoroughly characterize the transcriptional status of thousands of individual cells, enabling spatially resolved visualization and quantitative transcriptomic analysis in tissue sections, and allowing for an unbiased view of diverse cell populations, gene expression, and metabolic dysregulation within tissues [8, 9]. These technologies have been successfully applied to identify the cellular composition and spatial architecture in human cancers, such as cutaneous squamous cell carcinoma, lung cancer, and colorectal cancer [10].

Here, we combined scRNA-seq with ST to elucidate the spatiotemporal changes during the PCT for CSCC, along with matched normal cervix uteri. We utilized confocal microscopy to verify the subpopulations of fibroblasts, endothelial cells, and B cells during PCT using the premalignant and malignant tissues from the same patients diagnosed at different time points. Furthermore, we used multiple in vitro and in vivo experiments to characterize the expression and functional roles of a gene set, including CLDN1, ISG15, and PTGDS. We performed RNA sequencing to explore the signaling pathways mediated by CLDN1, ISG15, and PTGDS. The FGF1/FGFR1/PI3K/AKT signaling pathway was further validated with overexpression and inhibitor treatment. Together, these findings elucidate the tumorigenesis of CSCC, the cell subpopulations involved, the spatial niches in which they interact, and the gene networks they engage in within the tumor microenvironment, which offers a comprehensive view of the PCT of CSCC, suggesting that CLDN1, ISG15, and PTGDS are potential biomarkers associated with disease progression and diagnosis, as well as therapeutic targets for PCT.

## Results

### Single-cell transcriptomic atlas of normal, premalignant, and CSCC

**Fig. 1:**
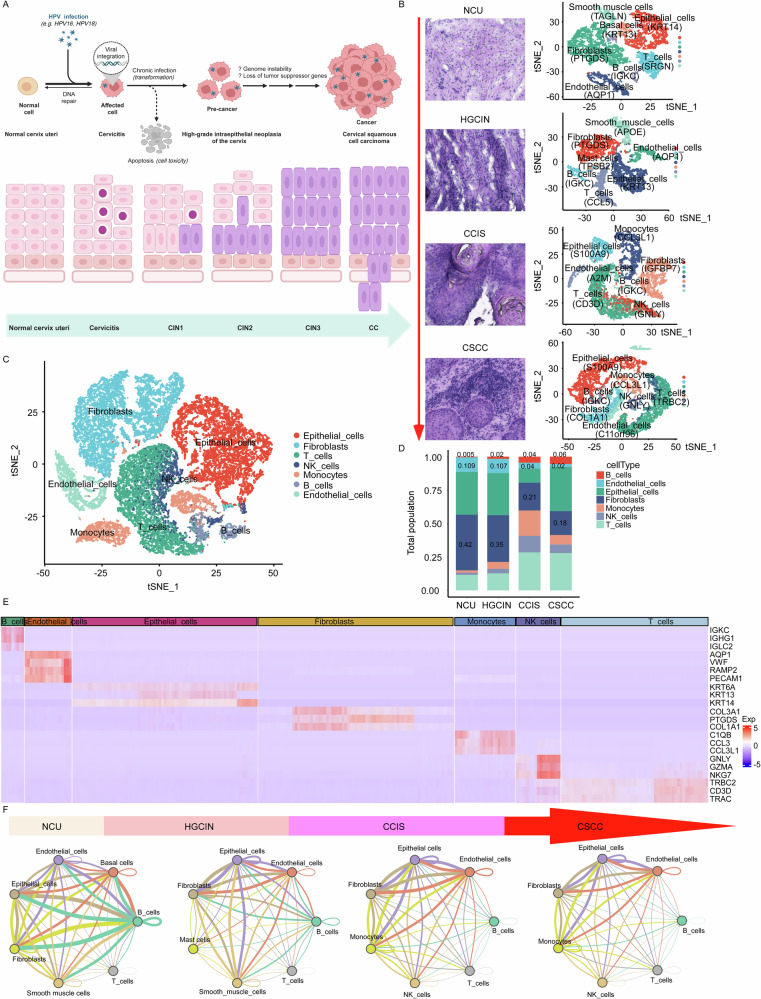
A single-cell atlas of premalignant and malignant lesions of the cervix uteri. **A** Schematic diagram of the progression cascade of CSCC development. **B**, **C** t-SNE plots of 31,812 high-quality cells visualizing cell-type clusters based on the expression of known marker genes in individual and integrated samples. **D** Bar plot of cell type distribution in different samples. **E** Heatmap of cell-type markers. The relative expression levels of genes across cells are presented and sorted by cell type. Cell-type marker genes were identified in an unbiased manner (Wilcoxon rank-sum test; FDR <0.1; fold change ≥2). **F** Diagram of the intercellular ligand-receptor interaction network in different samples, showing the number of ligand-receptor interactions detected between different cell types. Positive (from signaling cells to target cells) and reverse interactions are labeled, while autocrine signals are represented within each cell type. Nodes of the network diagram are color-coded by cell type, and edges are labeled and scaled by the number of interactions between the signaling and the target cells.

Over 120 HPV types have been identified, with nearly one-third infecting the squamous epithelium of the genital tract. HPVs are responsible for over 99% of cervical cancer cases [11]. Infection with high-risk HPVs is not sufficient but is necessary for cancer progression (International Agency for Research on Cancer Class I includes HPV types 16, 18, 31, 33, 35, 39, 45, 51, 52, 56, 58, and 59; Class 2A includes HPV68; and Class 2B includes HPV types 26, 53, 66, 67, 70, 73, and 82) [12]. Moreover, the lifetime risk of HPV infection is estimated to be 80%, with HPV16 and HPV18 responsible for nearly 70% of cervical cancers. E5, E6, and E7 are the main HPV oncoproteins involved in the initiation and progression of cervical cancer by targeting p53 and retinoblastoma (Rb) proteins to induce genomic instability. In addition, alterations in additional signaling pathways, such as cell cycle progression, apoptosis, telomere maintenance, and chromosomal stability are also essential for the malignant transformation (Fig. 1A). To construct the single-cell transcriptomic atlas of the cervix uteri in premalignant and tumor tissues, we collected samples representing a pathological cascade spanning from normal cervix uteri (NCU), high-grade cervical intraepithelial neoplasia (HGCIN), cervical carcinoma in situ (CCIS), to CSCC. The NCU, HGCIN, CCIS, and CSCC tissues were taken from the same patient infected with HPV16 (Table 1). The single cells were isolated without prior selection and processed using the 10x Chromium platform to generate single-cell RNA-seq data. A total of 39,120 cells were obtained by scRNA-seq, and 31,812 cells that passed quality control were retained for subsequent analysis (Table 2). Then, dimensionality reduction and unsupervised clustering of cells were performed using the Seurat software suite to identify distinct cell populations according to gene expression patterns [13], followed by batch effect correction among multiple samples. T-distributed stochastic neighbor embedding (t-SNE) was used to profile the cascade from NCU, HGCIN, and CCIS to CSCC. A total of 11 primary cell clusters were identified (Fig. S1A–H). The cells were further identified as 7 distinct cell types subsequently based on the known marker genes, including epithelial cells (8379, 26.34%), endothelial cells (2113, 6.64%), fibroblasts (8,846, 27.81%), and immune cells (B cells: 1032, 3.24%; T cells: 6673, 20.98%; monocytes: 2757, 8.67%; NK cells: 2012, 6.32%; total: 12,474, 39.21%) (Tables 3, 4), which we defined as the single-cell transcriptome atlas of human CSCC in premalignant and malignant lesions (Fig. 1B–D). The composition and proportion of cell types varied across samples. The main cell types in NCU and HGCIN samples are epithelial cells (32.50% and 31.47%, respectively) and fibroblasts (41.62% and 34.95%, respectively), while the immune cells accounted for the largest proportion (64.23% and 46.87%, separately) in CCIS and CSCC samples. This result was consistent with previous studies on the tumor immune microenvironment of cervical cancer, in which T and NK cells exhibited a marked enrichment in the tumor area [14, 15]. The endothelial cells progressively decreased in the CSCC cascade, which ranked from 10.86% (NCU), 10.68% (HGCIN), 4.41% (CCIS) to 2.20% (CSCC). Based on the clustering results, marker genes specific to each cluster were identified by a non-parametric test (Wilcoxon rank-sum test). Based on the marker gene expression, we found that the atlas predominantly consisted of non-immune cell lineages, including epithelial cells (top marker genes: KRTs, S100A8/9, and FABP5), endothelial cells (top marker genes: AQP1, RAMP2, PECAM1, VWF), and fibroblasts (top marker genes: PTGDS, COL1A1/COL3A1/COL1A2, and IGFBP5), as well as immune cell lineages, containing B cells (top marker genes: IGKC, IGHG1/3/2, and IGHA1), T cells (top marker genes: TRBC2/1, CD3C, and TRAC), NK cells (top marker genes: GNLY, GZMA/B, NKG7, and CD7), and monocytes (top marker genes: CCL3L1/CCL3/CCL4L2/CCL4, C1QB/A, IL1B, and HLA-DRA) (Fig. 1E, Fig. S1I). Multicellular organisms are open and complex systems composed of many different cell types. Intercellular communication, mediated through ligand-receptor interactions, is essential for regulating key biological processes, including development, differentiation, and inflammation. Cell communication analysis, also known as cell ligand-receptor interaction analysis, takes gene expression data of cell subsets as the research object. By obtaining the expression information of ligand and receptor genes in cells, the expression differences of ligand and receptor genes between cell types are compared, and the signal communication relationship between cell subsets is analyzed. It is of great significance to elucidate the complexity, diversity, and dynamics of cellular communication in biological processes. CellPhoneDB, a tool containing 978 curated proteins sourced from UniProt, Ensembl, PDB, and IUPHAR databases, was used for cell communication analysis. The single-cell-based network showed conserved cell types across lesions, which were mainly composed of epithelial cells, endothelial cells, and fibroblasts (Figs. 1F, S2). However, cell communication patterns varied across disease stages, such that the predominant cells in cell communication networks were B cells in NCU, epithelial cells in HGCIN, and epithelial cells, endothelial cells, as well as monocytes in CSCC (Figs. 1F, S2). Communication between B cells and other cells was progressively weakened during CSCC progression (Figs. 1F, S2). These results are consistent with previous research indicating that protumor interactions between epithelial cells and macrophages contribute to the transition from low-grade squamous intraepithelial lesions (LSIL) to high-grade squamous intraepithelial lesions (HSIL) [16]. We also collected NCU, HGCIN, and CSCC tissues from another patient infected with HPV58 and performed scRNA-seq (Table 1). Integrated analysis of single-cell data from patients infected with HPV16 and HPV58 showed that the alterations in the single-cell transcriptomic landscape closely resembled those observed in HPV16-infected patients (Fig. S3).

**Table 1:** Clinical characteristics of patients.

Patient	Age	HPV	Pathology	Differentiation	Invasion	Positive lymph node (n/N)
JGX	51	16	CSCC	High-medium	<1/3–2/3	0/24
SRL	49	16	CSCC	Medium-low	>2/3	3/23
LHL	43	58	CSCC	Medium	1/3–2/3	0/26

**Table 2:** CellRanger analyzed cell information.

Sample ID	Estimated number of Cells	Mean reads per Cell	Median UMI counts per Cell	Median genes per Cell	Total genes detected
NCU	9345	49,484	7961	2375	29,463
HGCIN	8641	55,585	8944	2594	30,320
CCIS	10,542	56,538	5362	1842	29,828
CSCC	10,592	44,593	4520	1568	29,462

**Table 3:** Numbers of different cell types in different samples (Single).

Cell type	NCU	HGCIN	CCIS	CSCC
Epithelial cells	1849	2076	858	2831
Endothelial cells	752	725	384	806
Fibroblasts	2609	1807	1817	1057
Smooth muscle cells	414	827		
Basal cells	322			
B cells	310	470	355	517
T cells	928	1028	2551	2559
Mast cells		109		
Monocytes			1652	609
NK cells			1072	518
Total cells	7184	7042	8689	8897

**Table 4:** Numbers of different cell types in different samples (Integrated).

Cell type	NCU	HGCIN	CCIS	CSCC
Epithelial cells	2302	2216	922	2939
Endothelial cells	782	752	383	196
Fibroblasts	2990	2461	1803	1592
B cells	35	115	391	491
T cells	831	894	2469	2479
Monocytes	124	362	1644	627
NK cells	120	242	1077	573
Total cells	7184	7042	8689	8897

### Spatiotemporal architecture of normal, premalignant, and CSCC

**Fig. 2:**
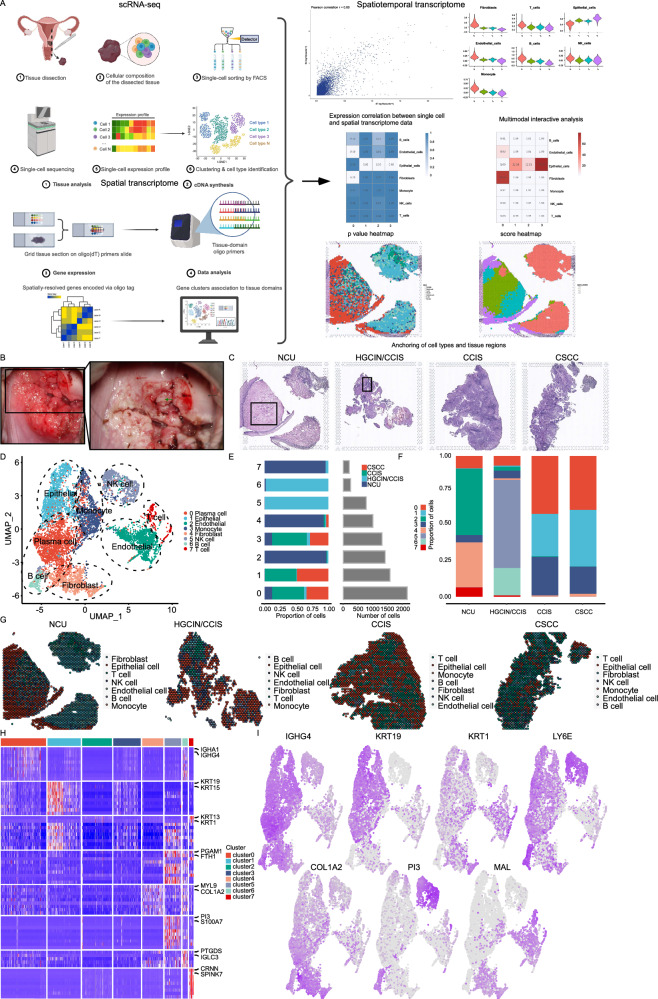
Spatiotemporal architecture of normal, premalignant, and cervical squamous cell carcinoma tissues. **A** Schematic diagram of single-cell RNA sequencing, spatial transcriptomics, and integrative analysis. **B** Images of normal (black arrow), premalignant (red arrow), and CSCC tissues (green arrow). **C** H&E staining of different tissues. **D** UMAP plot of cell populations in integrated samples. **E** Proportions and numbers of cells in different tissues. **F** Relative proportions of different cell types in different tissues. **G** Spatial distribution of different cell types in different tissues. **H** Heatmap of marker genes in each cell type. **I** Expression of marker genes in the corresponding cell types.

Spatial transcriptomics is a technique for analyzing spatially resolved transcriptomic data, which profiles all mRNA in a single tissue section, thereby enabling the identification and differentiation of actively expressed functional genes within a defined tissue region. Morphological information of tissue sections was obtained by H&E staining and microscopic imaging. After spatial transcriptome analysis of the tissue sections, the expression of each gene at any position on the sections could be clearly determined. Therefore, spatial transcriptomics integrates two types of information, gene expression and spatial location, enabling precise gene localization. To comprehensively characterize the spatial architecture of normal, premalignant, and CSCC, we collected fresh NCU, HGCIN, CCIS, and CSCC tissues from the same patient infected with HPV16 and applied ST profiling using the 10x Genomics Visium platform (Tables 1, 5). A total of 8668 spots and 92,186 genes were obtained by ST sequencing of four samples. Gene expression is spatially and temporally specific. ScRNA-seq primarily investigates gene expression over time and systematically identifies cell subsets within tissues, but it does not capture their spatial organization, which impedes our comprehension of tissue architecture and intercellular interactions. The application of ST makes it possible to analyze data from a spatial perspective and study gene expression within spatial context. Given the inherent limitations of both scRNA-seq and ST, it is of great significance to integrate the two data modalities by combining scRNA-seq data with ST data to analyze gene expression in space and time, thereby obtaining a comprehensive spatiotemporal architecture (Fig. 2A). To analyze the expression correlation between single-cell and spatial transcriptome genes, we conducted Pearson correlation analysis between scRNA-seq and ST gene expression profiles. When scRNA-seq and ST were performed on the same sample, there was a positive correlation in overall gene expression (Fig. S4A). Moreover, the Seurat package was used to integrate single-cell data and spatial transcriptome data, and the two data sets were integrated based on anchor points, allowing annotation information to be transferred from the reference data set (single-cell data set) to the query dataset (spatial transcriptome data set). The cell types of each spot in the spatial transcriptome were analyzed. This analysis revealed a similar single-cell atlas and spatiotemporal pattern of cell-type distribution to the scRNA-seq data, in which epithelial cells and fibroblasts were predominant in NCU and HGCIN samples, while immune cells showed a higher enrichment in CCIS and CSCC samples (Figs. 2B–G, S4B, C). Annotated genes for each cell type were visualized (Fig. 2H, I). Together with the scRNA-seq data and previously published studies, these results indicate the high reliability and consistency of these findings.

**Table 5:** SpaceRanger analysis information statistics.

Sample ID	Number of spots under Tissue	Mean reads per spot	Median UMI counts per Spot	Median genes per spot	Total genes detected
CSCC	1939	98414.2485817432	7650	3305	23,104
HGCIN	1232	176651.452922078	17,944	4779	23,416
CCIS	2603	81325.4479446792	6069	2676	23,398
NCU	2894	68355.4467864547	1831.5	895.5	22,268

### Cell subpopulation analysis profiles reveal immunosuppression during CSCC progression

#### A GNLY-COL3A1 CAF subset is identified in the PCT of CSCC

**Fig. 3:**
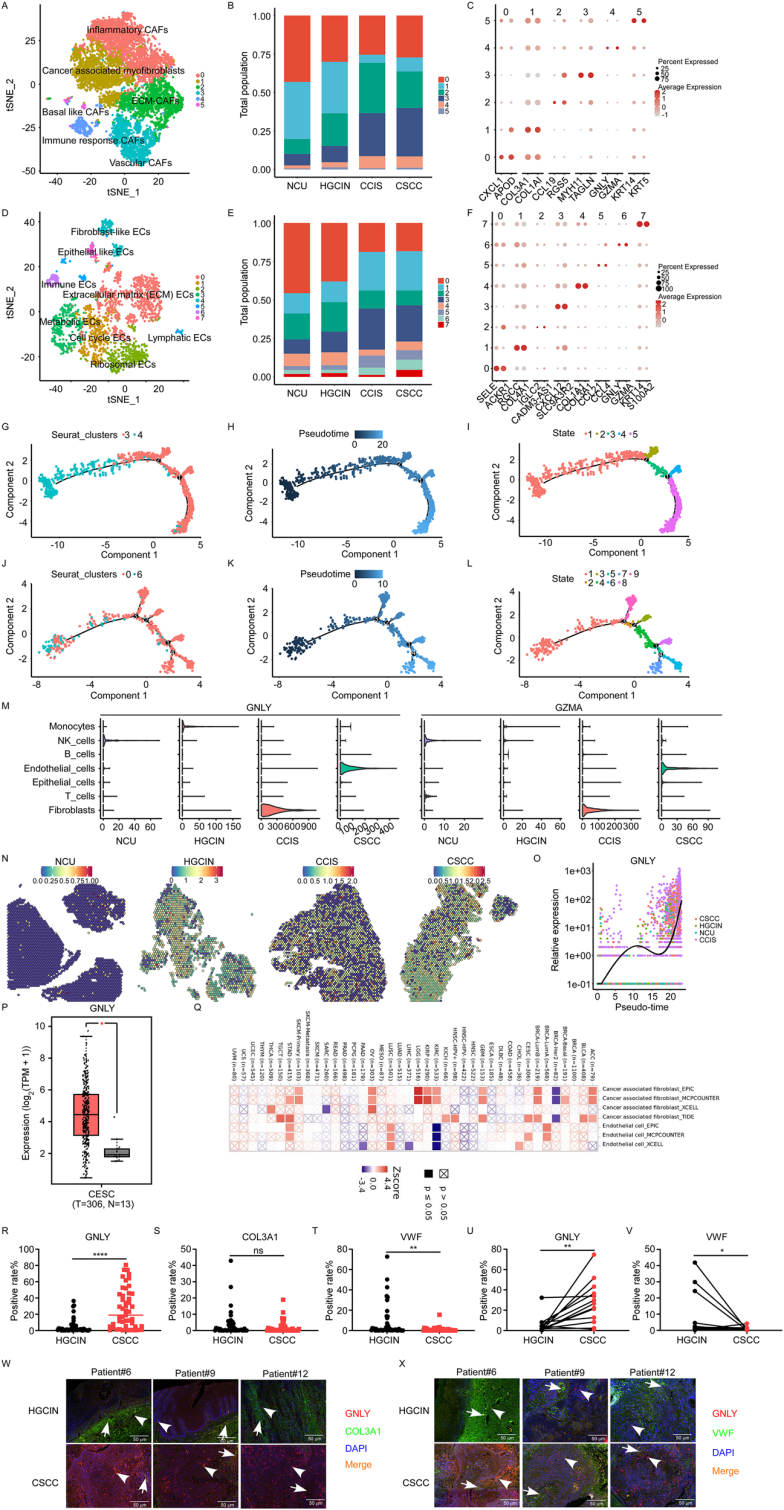
GNLY-COL3A1 fibroblasts and GNLY-VWF endothelial cells are associated with the precancer-to-cancer transition of CSCC. **A** t-SNE representation of six subpopulations identified within fibroblasts. **B** Histogram of fibroblast subpopulation proportions in different samples. **C** Marker gene bubble plot of fibroblast subpopulations in different samples (top 2). **D** t-SNE representation of eight subpopulations identified within endothelial cells. **E** Histogram of endothelial cell subpopulation proportions in different samples. **F** Marker gene bubble plot of endothelial cell subpopulations in different samples (top 2). **G**–**I** Pseudotime analysis of vascular CAFs and immune response CAFs with different states. **J**–**L** Pseudotime trajectories of extracellular matrix endothelial cells and immune response endothelial cells with distinct states. **M** Violin plots of GNLY and GZMA expression in different tissues. **N** Spatial distribution and expression of GNLY in different tissues. **O** Pseudotime expression dynamics of GNLY in different tissues. **P** GNLY expression in normal cervix uteri and cervical cancer tissues based on the GEPIA database. Tumor samples were derived from the TCGA-CESC cohort (*n* = 306), and normal tissues were obtained from matched TCGA and GTEx data (*n* = 13), analyzed via GEPIA. Samples with incomplete clinical information were excluded where applicable. **Q** Correlation analysis of GNLY expression with fibroblasts and endothelial cells using the TIMER2.0 database. **R**–**T** Positive staining rates of GNLY, COL3A1, and VWF in HGCIN and CSCC patient tissues. **U**, **V** Positive staining rates of GNLY and VWF in paired HGCIN and CSCC tissues from the same patients. **W** Representative confocal microscopy images of GNLY and COL3A1 in tissues from the same patients who progressed from HGCIN to CSCC (*n* = 13). Scale bars: 50 µm. **X** Representative confocal microscopy images of GNLY and VWF in tissue samples from patients who transitioned from HGCIN to CSCC (*n* = 13). Scale bars: 50 µm. Statistical significance was determined by Student’s *t*-test (**R**–**V**). The *p-*value <0.05 was considered statistically significant (*p* < 0.05: *, *p* < 0.01: **, *p* < 0.001: ***, *p* < 0.0001: ****).

Single-cell RNA sequencing and ST sequencing data revealed that the proportions of endothelial cells (ECs) and fibroblasts decreased, whereas those of B cells increased during CSCC progression (Figs. 1D, 2E, F). Therefore, we analyzed the subpopulations of these cells. The fibroblast subpopulations comprised six CAF subtypes: cancer-associated myofibroblasts (Myo-CAFs), inflammatory CAFs (I-CAFs), basal-like CAFs (BL-CAFs), extracellular matrix CAFs (ECM-CAFs), vascular CAFs (V-CAFs), and immune response CAFs (IR-CAFs) (Fig. 3A). The percentages of I-CAFs (top 2 marker genes: CXCL1, APOD) and Myo-CAFs (top 2 marker genes: COL3A1, COL1A1) showed a decreasing trend, while the V-CAFs (top 2 marker genes: MYH11, TAGLN) and IR-CAFs (top 2 marker genes: GNLY, GZMA) showed an increasing trend (Fig. 3B, C). The biological processes of V-CAFs were mainly enriched in collagen metabolic process and blood vessel development (Fig. S5A, B). Kyoto Encyclopedia of Genes and Genomes (KEGG) pathway showed enrichment in protein digestion and absorption, focal adhesion, and the PI3K/AKT signaling pathway (Fig. S5C). Moreover, the upregulated top 2 marker genes TAGLN (transgelin) and MYH11 (myosin heavy chain 11) had significantly higher expression in cervical cancer tissues than in normal tissues, while the downregulated top 2 marker genes PTGDS (prostaglandin D2 synthase) and SFRP4 (secreted frizzled related protein 4) showed lower expression levels (Fig. S5D). The biological processes of IR-CAFs were enriched in the TNF-mediated signaling pathway, T cell receptor signaling pathway, and the immune response (Fig. S5E, F). KEGG pathway analysis showed genes in IR-CAFs were enriched in the immune system, cytokine signaling in the immune system, PD-1 signaling, TCR signaling, as well as signaling by interleukins (Fig. S5G). The upregulated top 2 marker genes, GNLY (granulysin) and GZMA (granzyme A) were highly expressed in cervical cancer tissues (Fig. S5H). GNLY was highly expressed in CSCC tissues and promoted cell proliferation and colony formation of CSCC cells in vitro (Fig. S5I–M).

The endothelial subpopulations included extracellular matrix endothelial cells (ECM-ECs), cell cycle ECs (CC-ECs), ribosomal ECs (R-ECs), metabolic ECs (M-ECs), fibroblast-like ECs (FL-ECs), lymphatic ECs (L-ECs), immune response ECs (IR-ECs), and epithelial-like ECs (EL-ECs) (Fig. 3D). The proportion of ECM-ECs (top 2 marker genes: SELE, ACKR1) decreased, whereas IR-ECs (top 2 marker genes: GNLY, GZMA) increased progressively during CSCC progression (Fig. 3E, F). Further analysis showed that ECM-ECs marker genes were mainly involved in collagen catabolic process, reverse cholesterol transport, positive regulation of epithelial-mesenchymal transition (EMT), negative regulation of ECs proliferation, and blood vessel development (Fig. S6A, B). These genes were enriched in the AGE-RAGE signaling pathway with diabetic complications, ECM-receptor interaction, protein digestion and absorption, PI3K/AKT signaling pathway, and focal adhesion (Fig. S6C). We analyzed the expression of top marker genes in normal and CSCC tissues in the GEPIA database. The results showed that SELE (selectin E) and ACKR1 (atypical chemokine receptor 1) were significantly downregulated in CSCC tissues (Fig. S6D). SELE encodes E-selectin, which is reported as a cell-surface glycoprotein playing a role in immunoadhesion, negatively regulating neutrophil transmigration through alterations in endothelial plasticity [17]. The biological process and signaling pathways of IR-ECs were mainly involved in immune response (Fig. S6E–H). These results indicate that dysregulated fibroblasts and endothelial cell subpopulations may play key roles in immune regulation during PCT of CSCC.

Then we analyzed the pseudotime trajectories and RNA velocity of V-CAFs, IR-CAFs, ECM-ECs, and IR-ECs. The results showed that IR-CAFs and IR-ECs transitioned toward V-CAFs and ECM-ECs with several cell states (Figs. 3G–L, S6I, J). This analysis revealed that the immune response was progressively attenuated during PCT of CSCC. The expression of the top two marker genes in IR-CAFs and IR-ECs was analyzed, and the results showed that GNLY and GZMA were mainly expressed in fibroblasts and endothelial cells, displaying an increasing trend during CSCC progression (Fig. 3M). Moreover, GNLY expression was higher than that of GZMA (Fig. 3M). The spatial distribution and expression of GNLY were visualized, further confirming its increased expression (Fig. 3N). Pseudotime analysis further demonstrated that GNLY expression increased in CSCC progression (Fig. 3O). GNLY expression in NCU and cervical cancer was analyzed in the GEPIA database, which showed that GNLY levels were significantly elevated in cervical cancer tissues compared with normal tissues (Fig. 3P). Correlation analysis revealed that GNLY expression had a positive correlation with CAFs and ECs in cervical cancer (Fig. 3Q). Finally, we performed multiplex immunohistochemistry (mIHC) on the precancer lesions diagnosed previously and cancer tissues developed ultimately obtained from the same patients. The results showed that the positive rate of GNLY increased, whereas VWF (an endothelial cell marker) decreased in both total and paired CSCC tissues compared to total and paired HGCIN tissues (Fig. 3R–V). These findings indicate that the proportions of GNLY-IR-CAFs and GNLY-IR-ECs were significantly enriched during the PCT of CSCC (Figs. 3W, X, S7). Collectively, these results suggest that GNLY-IR-CAFs and GNLY-IR-ECs may serve as biomarkers for PCT of CSCC.

#### An IGFBP7 progenitor cell subset was verified during the PCT of CSCC

**Fig. 4:**
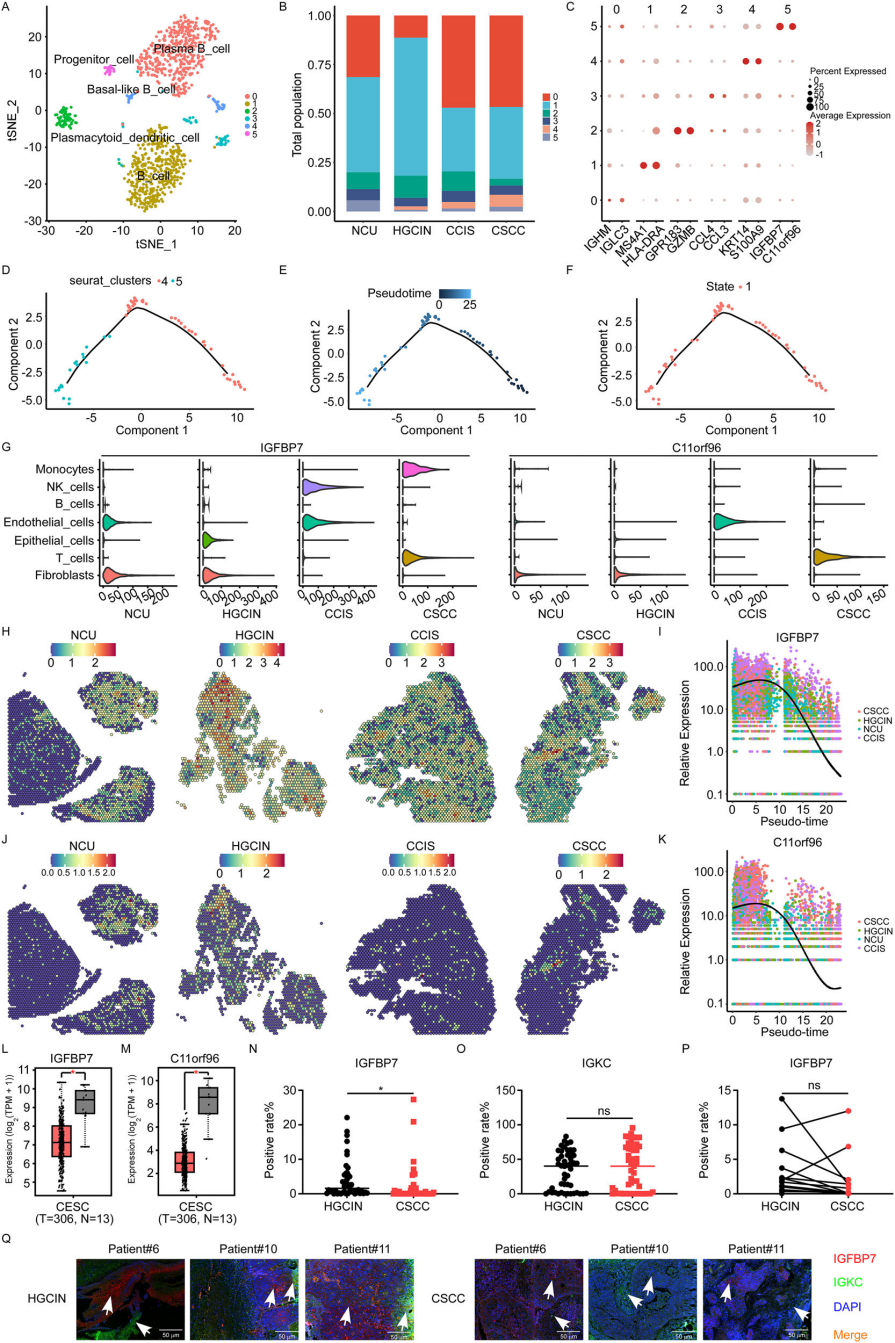
IGFBP7-B cells are associated with the precancer-to-cancer transition of CSCC. **A** t-SNE representation of subpopulations generated from B cells. **B** Histogram of B-cell subpopulation proportions in different samples. **C** Bubble plot of the top two marker genes for each B-cell subpopulation across samples. **D**–**F** Pseudotime trajectories of basal-like B cells and progenitor cells with different states. **G** Violin plots of IGFBP7 and C11orf96 expression in different tissues. **H**, **J** Spatial distribution and expression of IGFBP7 and C11orf96 in different tissues, respectively. **I**, **K** Pseudotime expression dynamics of IGFBP7 and C11orf96 in different tissues, respectively. **L**, **M** Expression of IGFBP7 and C11orf96 in normal cervical tissues and cervical cancer tissues based on the GEPIA database. Tumor samples were derived from the TCGA-CESC cohort (*n* = 306), and normal tissues were derived from matched TCGA and GTEx data (*n* = 13), analyzed via GEPIA. Samples with incomplete clinical information were excluded where applicable. **N**, **O** Positive staining rates of IGFBP7 and IGKC in HGCIN and CSCC tissues. **P** Positive rates of IGFBP7 in paired HGCIN and CSCC tissues from patients diagnosed at different time points. **Q** Representative laser confocal images of IGFBP7 and IGKC in tissues from the same patients who progressed from HGCIN to CSCC (*n* = 13). Scale bars: 50 µm. Statistical significance was determined by Student’s t-test (N-P). The *p-*value < 0.05 was considered statistically significant (*p* < 0.05: *).

Immune cells are essential in the tumor microenvironment (TME) and interact with tumor cells to regulate tumorigenesis, tumor progression, and therapeutic response, either directly or indirectly. To explore the diversity of immune cells involved in the development of CSCC, we analyzed B-cell subpopulations. B cells were divided into five subpopulations: progenitor cells (top 2 marker genes: IFITM3, IGFBP7), B cells (top 3 marker genes: HLA-DRA, MS4A1, CD69), plasma B cells (top 2 marker genes: IGHA1, IGLC3), basal-like B cells (top 3 marker genes: S100A8, S100A9, KRT14), and plasmacytoid dendritic cells (top 2 marker genes: GPR183, GZMB) (Fig. 4A, C). Moreover, the proportion of basal-like B cells increased during CSCC progression, whereas progenitor cells decreased (Fig. 4B). The biological processes of basal-like B cells were enriched in SRP-dependent cotranslational targeting membrane, translational initiation, and viral transcription (Fig. S8A, B). KEGG pathway analysis revealed enrichment in ribosome and oxidative phosphorylation pathways (Fig. S8C). The expression of the top basal-like B cell marker gene S100A9 (S100 calcium-binding protein A9) was upregulated in cervical cancer (Fig. S8D). The biological processes of progenitor cells were enriched in collagen process and ECM organization (Fig. S8E, F). KEGG pathways analysis revealed enrichment in focal adhesion, protein digestion and absorption, ECM-receptor interaction, TGF-β signaling pathway, as well as PI3K/AKT signaling pathway (Fig. S8G). The expression of progenitor cell marker genes IGFBP7 (insulin-like growth factor binding protein 7) and C11orf96 (chromosome 11 open reading frame 96) was downregulated in cervical cancer tissues (Figure S8H). Pseudotime trajectory analysis and RNA velocity indicated that progenitor cells transitioned toward basal-like B cells during CSCC progression (Figs. 4D–F, S8I). This transformation reflected the process of B-cell development and differentiation. The expression levels of IGFBP7 and C11orf96 were low in B cells (Fig. 4G). The spatial expression levels of IGFBP7 and C11orf96 decreased during the PCT (Fig. 4H, J). Pseudotime analysis confirmed these findings (Fig. 4I, K). Cervical cancer tissues exhibited significantly reduced expression of IGFBP7 and C11orf96 compared with those in NCU tissues (Fig. 4L, M). We verified IGFBP7-positive progenitor cells by mIHC in precancer and cancer tissues from the same patients diagnosed at different time points. The results showed a significant decrease in the IGFBP7 positivity rate in CSCC tissues compared with HGCIN tissues (Figs. 4N–Q, S8J). These findings suggest suppressed mature B-cell differentiation and impaired immune responses during the PCT of CSCC.

#### PTGDS, CLDN1, and ISG15 are potential biomarkers for PCT of CSCC

**Fig. 5:**
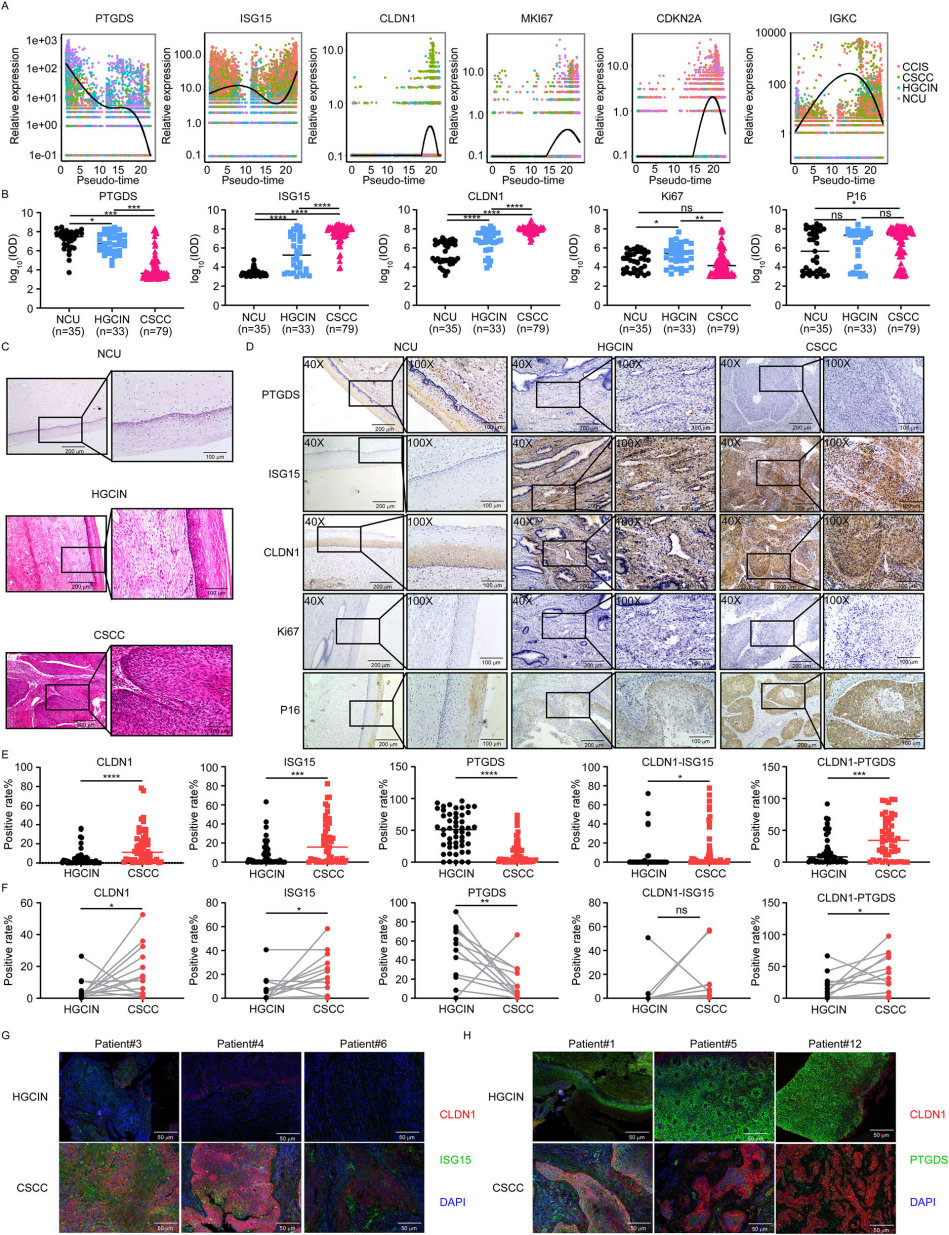
Expression of CLDN1, PTGDS, and ISG15 in the precancer-to-cancer transition of cervical cancer. **A** Pseudotime expression dynamics of PTGDS, ISG15, CLDN1, MKI67, CDKN2A, and IGKC in different tissues. **B** Statistical analysis of IHC staining levels of PTGDS, ISG15, CLDN1, Ki67, and P16 in precancer and cancer tissues. **C** Representative H&E staining images of normal, precancer, and tumor tissues. Scale bars: 200 µm (low magnification) and 100 µm (high magnification). **D** Representative images of IHC staining with different markers in different tissues. Scale bars: 200 µm (low magnification) and 100 µm (high magnification). **E** Statistical analysis of mIHC positivity rates of CLDN1, ISG15, and PTGDS in precancer and cancer tissues from the same patients diagnosed at different time points (*n* = 13). **F** Statistical analysis of mIHC positive rates of CLDN1, ISG15, and PTGDS in paired precancer and cancer tissues from the same patients diagnosed at different time points (n = 13). **G**, **H** Representative images of CLDN1/ISG15 and CLDN1/PTGDS mIHC staining, respectively. Scale bars: 50 µm. Statistical significance was determined by one-way ANOVA (**B**), Student’s *t*-test (**E**, **F**). The *p-*value < 0.05 was considered statistically significant (*p* < 0.05: *, *p* < 0.01: **, *p* < 0.001: ***, *p* < 0.0001: ****).

We investigated the potential biomarkers of PCT in CSCC by analyzing dysregulated genes using scRNA-seq and spatial transcriptomics data (Fig. S9A, B). The results showed that IGKC, PTGDS, and CLDN1 exhibited similar expression patterns in both analyses. Subsequently, we examined the spatial distribution and expression of these genes, including ISG15, CDKN2A, and MKI67, across different tissues. The expression levels of IGKC, CLDN1, ISG15, CDKN2A, and MKI67 increased during PCT of CSCC, while PTGDS expression decreased (Fig. S9C, D). Moreover, pseudotime analysis further confirmed the same expression trends (Fig. 5A). Subsequently, we analyzed the expression of these genes using the GEPIA database. The results indicated that CLDN1 and ISG15 had a higher expression level in cervical cancer tissues compared to normal tissues, while PTGDS showed an opposite expression trend (Fig. S10A–C). IGKC expression showed no significant difference (Fig. S10D). We further analyzed CLDN1, ISG15, and PTGDS expression in the TCGA database, and the results showed that PTGDS was downregulated, whereas ISG15 was upregulated in cervical cancer tissues compared to normal tissues (Fig. S10E). Additionally, high PTGDS expression was associated with longer survival in cervical cancer patients (Fig. S10F). Furthermore, we analyzed the correlation between PTGDS, IGKC, CLDN1, and ISG15 expression and clinical characteristics in cervical cancer patients. The results showed that IGKC, CLDN1, and ISG15 expression significantly differed among different histological types of cervical cancer. ISG15 and PTGDS expression varied significantly among different sample types. ISG15 expression showed a negative correlation with patients’ age at initial pathological diagnosis, CLDN1 expression correlated positively with the total number of premalignant lesions, whereas PTGDS expression correlated negatively with it (Fig. S10G). These results suggest that CLDN1, PTGDS, and ISG15 may serve as biomarkers associated with PCT of CSCC.

Next, we validated the expression of PTGDS, CLDN1, and ISG15 in patient-derived samples. First, we measured the relative mRNA expression of PTGDS, CLDN1, IGKC, and ISG15 in patients’ serum samples, using SCCA (squamous cell carcinoma antigen) as a positive control. The results showed that PTGDS expression was significantly lower in the serum of patients with precancerous lesions, while CLDN1, ISG15, IGKC, and SCCA showed no significant changes (Fig. S11A). Next, we used IHC staining to verify the expression of PTGDS, CLDN1, and ISG15 in normal, precancer, and cancer tissues, with P16 and Ki67 as positive controls. The results showed that CLDN1 and ISG15 expression increased during PCT of CSCC, whereas PTGDS expression decreased. However, Ki67 expression increased in precancer tissues but decreased in cancer tissues, while P16 expression increased in cancer tissues (Fig. 5B–D). To assess the diagnostic and prognostic potential of PTGDS, CLDN1, and ISG15 expression during PCT of CSCC. We collected paired precancerous and cancer tissues from the same patients diagnosed at different time points and examined PTGDS, CLDN1, and ISG15 expression using mIHC. The results showed that PTGDS, CLDN1, ISG15, CLDN1-PTGDS, and CLDN1-ISG15 expression changed significantly between precancer and cancer tissues (Fig. 5E). Moreover, PTGDS, CLDN1, ISG15, and CLDN1-PTGDS expression exhibited differences in paired tissues from the same patient (Figs. 5F–H, S11B, C). PTGDS exhibited the highest positive detection rate (92.31%) among patients, followed by the CLDN1-ISG15 (76.92%), CLDN1 and CLDN1-PTGDS (69.23%), and ISG15 (61.54%) (Table 6). The findings indicate that PTGDS, CLDN1, and ISG15 could serve as potential biomarkers for the PCT of CSCC in clinical settings.

**Table 6:** Positive rates of PTGDS, CLDN1, and ISG15 in PCT of CSCC from the same patient (Fold change > 1.5).

Name	Positive rate
PTGDS	92.31%
CLDN1	69.23%
ISG15	61.54%
CLDN1-PTGDS	69.23%
CLDN1-ISG15	76.92%

#### PTGDS, CLDN1, and ISG15 play important roles in CSCC progression by regulating the FGF1/FGFR1/PI3K/AKT signaling pathway

**Fig. 6:**
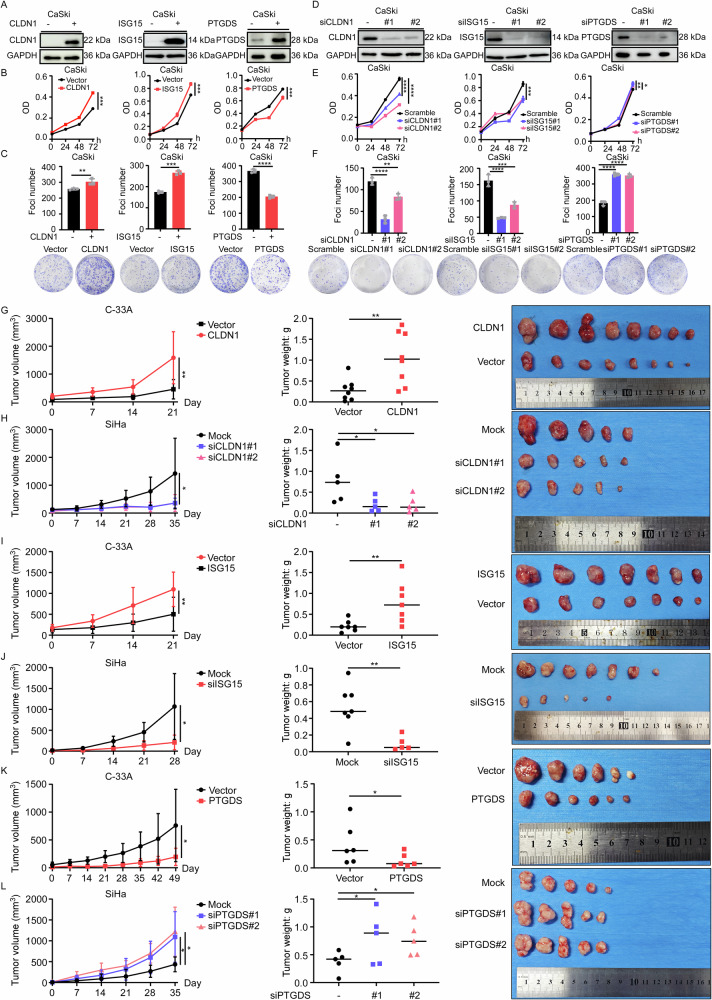
Function of CLDN1, ISG15, and PTGDS in cervical cancer cells and cell-derived xenograft models. **A** Overexpression of CLDN1, ISG15, and PTGDS in CaSki cells. **B** Proliferation of CLDN1, ISG15, and PTGDS overexpressed cells was assessed by MTT assay. **C** Colony formation of CLDN1-, ISG15-, and PTGDS-overexpressed cells was evaluated by crystal violet staining. **D** Knockdown of CLDN1, ISG15, and PTGDS in CaSki cells. **E** Proliferation of CLDN1, ISG15, and PTGDS-knockdown cells was verified by MTT assay. **F** Colony formation of CLDN1, ISG15, and PTGDS knockdown cells was evaluated by crystal violet staining. **G** Tumor volume (mm^3^), tumor weight, and tumors in CLDN1-overexpressed CDX mouse model. Tumor volume was measured once a week for 3 weeks. **H** Tumor volume (mm^3^), tumor weight, and tumors in CLDN1 knockdown CDX mouse model. Tumor volume was measured once a week for 5 weeks. **I**, **J** Tumor volume (mm^3^), tumor weight, and tumors in ISG15 overexpressed (**I**) and knockdown (**J**) CDX mouse models. Tumor volume was measured once a week for 3 weeks (**I**) and 4 weeks (**J**). **K, L** Tumor volume (mm^3^), tumor weight, and tumors in PTGDS overexpressed (**K**) and knockdown (**L**) CDX mouse models. Tumor volume was measured once a week for 7 weeks (**K**) and 5 weeks (**L**). Statistical significance was determined by one-way ANOVA (**E**, **F**, **H**, **L**) and Student’s *t*-test (**B**, **C**, **G**, **I**–**K**). The *p-*value < 0.05 was considered statistically significant (*p* < 0.05: *, *p* < 0.01: **, *p* < 0.001: ***, *p* < 0.0001: ****).

To explore the function of PTGDS, CLDN1, and ISG15 in CSCC progression, we constructed cell lines with overexpression and knockdown of these genes. The results showed that upregulation of CLDN1 significantly promoted cell proliferation and colony formation, whereas CLDN1 knockdown markedly suppressed cell proliferation and colony formation (Figs. 6A–F, S12A–F). ISG15 overexpression markedly enhanced cell proliferation and colony formation, whereas ISG15 downregulation significantly impaired cell growth (Figs. 6A–F, S12G-L). PTGDS overexpression reduced cell proliferation and colony formation, while PTGDS knockdown increased in these processes (Figs. 6A–F, S12M–R). These results suggest that CLDN1 and ISG15 act as oncogenic drivers in CSCC progression, whereas PTGDS functions as a tumor suppressor.

To elucidate the mechanisms of PTGDS, CLDN1, and ISG15 in CSCC progression, we performed RNA sequencing on PTGDS-, CLDN1-, and ISG15- overexpressed cells. The results showed that the overlapping differentially expressed genes among the three groups included IGFBP3, HDAC9, RRN3, and FGF1 (Fig. S13A). The enriched signaling pathways were predominantly associated with the PI3K/AKT signaling pathway (Fig. S13B). Moreover, the scRNA-seq data also revealed that the PI3K/AKT signaling pathway was enriched in fibroblasts, endothelial cells, and B cells (Fig. S13C–E). The spatial expression of PIK3CA, AKT1/2/3, and MTOR increased during the PCT (Fig. S13F–J).

To explore the therapeutic potential of PTGDS, CLDN1, and ISG15 in cervical cancer, we established several cell-derived xenograft (CDX) mouse models. The results demonstrated that CLDN1 overexpression significantly increased tumor volume and tumor weight, whereas CLDN1 knockdown markedly decreased both (Fig. 6G, H). Moreover, ISG15 overexpression significantly promoted tumor growth (Fig. 6I). ISG15 knockdown suppressed tumor volume and tumor weight (Fig. 6J). PTGDS overexpression markedly inhibited tumor growth, while PTGDS downregulation led to a marked increase in tumor volume and tumor weight (Fig. 6K, L). These findings collectively highlight the potential of PTGDS, CLDN1, and ISG15 as therapeutic targets in cervical cancer.

#### Inflammatory CAFs promote CSCC progression primarily through stabilizing FGF1 via ISG15

**Fig. 7:**
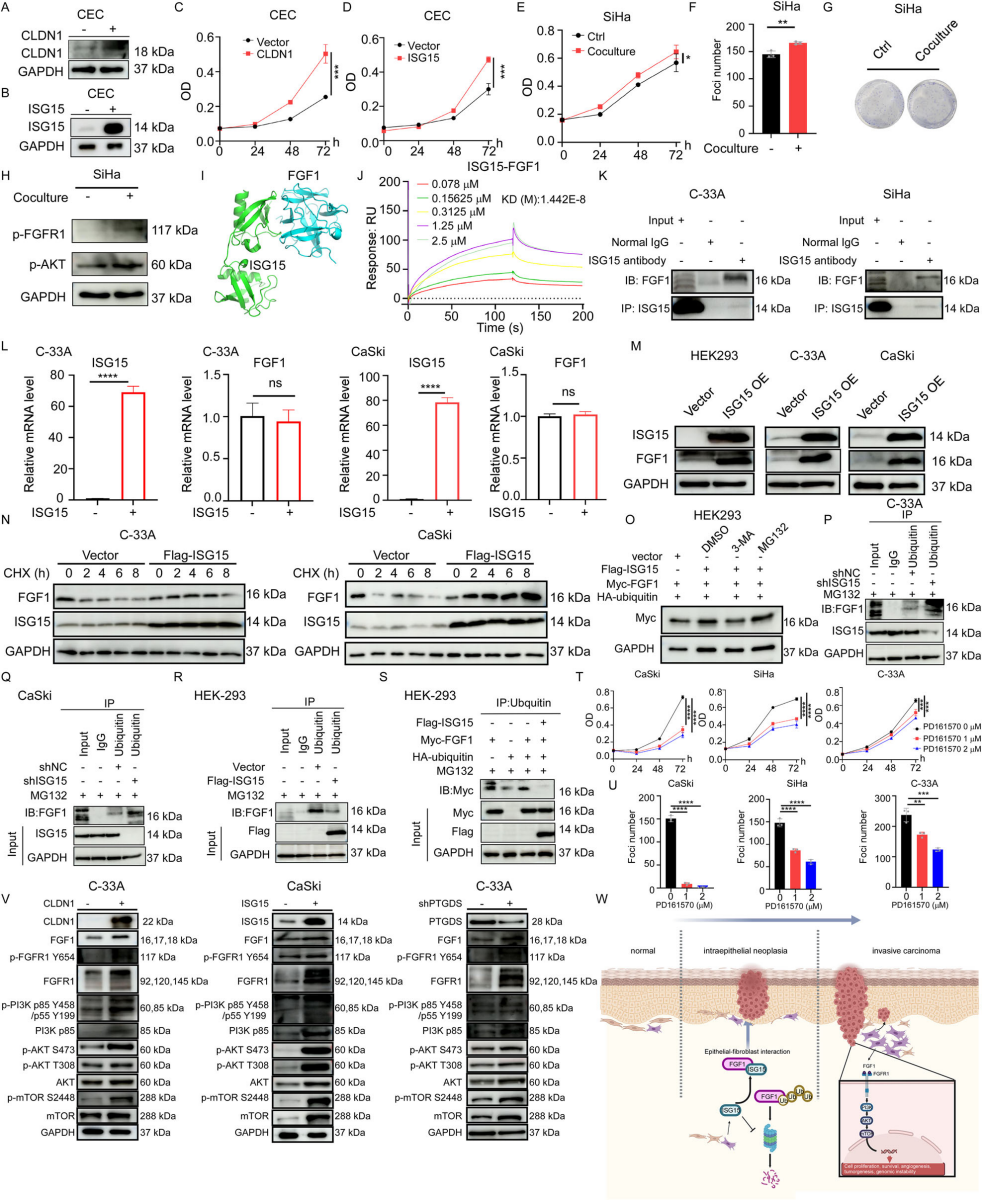
ISG15 binds to FGF1 and enhances FGF1 stability. **A** CLDN1 expression was detected in CLDN1-overexpressed human cervical epithelial cells (CECs) by Western blotting. **B** ISG15 expression was verified in ISG15-overexpressed CECs by Western blotting. **C** Proliferation of CLDN1-overexpressed human CECs was assessed by MTT assay. **D** Proliferation of ISG15-overexpressed human CECs was detected by MTT assay. **E** Proliferation of SiHa cells with or without human cervical fibroblasts co-culture was assessed by MTT assay. **F**, **G** Colony formation of SiHa cells with or without coculture human cervical fibroblasts. **H** Western blot analysis was performed to determine the levels of p-FGFR1 (Y654) and p-AKT (S473) in SiHa cells under conditions with or without cervical fibroblasts co-culture. **I** Molecular docking model indicated the binding between FGF1 and ISG15. (docking score = -242.92; confidence score: 0.8651). **J** Interaction of FGF1 with ISG15 was verified by SPR assay (KD (M) = 1.442E-8, FGF1 concentration: 0.078, 0.15625, 0.3125, 1.25, 2.5 µM). **K** Immunoprecipitation assay of FGF1 and ISG15 in C-33A and SiHa cells. **L** Real-time PCR analysis of FGF1 mRNA levels after ISG15 overexpression. **M** Western blotting assay of FGF1 protein levels following ISG15 overexpression in HEK293, C-33A, and CaSki cells. **N** C-33A and CaSki cells transfected with empty vector or ISG15-encoding plasmid were treated with cycloheximide (CHX) for the indicated times. **O** Western blotting was used to measure FGF1 expression following 3-MA and MG132 treatment. **P**, **Q** Lysates from cells expressing control shRNA or MG132-treated ISG15 knockdown cells were immunoprecipitated to assess FGF1-ISG15 interaction. **R** Lysates from cells expressing an empty vector or ISG15, MG132-treated cells were immunoprecipitated to detect the binding affinity. **S** Total lysates derived from HEK293 cells co-transfected with different plasmids for 48 h and subsequently treated with MG132 for 6-8 h were immunoprecipitated to detect the binding affinity. **T** Proliferation of cervical cancer cells treated with graded concentrations of PD161570 was assessed by MTT assay. **U** Colony formation assays of cervical cancer cells treated with different concentrations of PD161570. **V** Expression of key markers in the FGF1/FGFR1/PI3K/AKT signaling pathway was examined by Western blotting in CLDN1-, ISG15-overexpressed, and PTGDS-knockdown cells. **W** The diagram of the interaction between inflammatory cancer-associated fibroblasts and tumor cells in the progression of cervical squamous cell carcinoma. Statistical significance was determined by Student’s *t*-test (**C**–**F**, **L**) and one-way ANOVA (**T**, **U**). The *p-*value < 0.05 was considered statistically significant (*p* < 0.05: *, *p* < 0.01: **, *p* < 0.001: ***, *p* < 0.0001: ****).

CAFs promote tumor progression by releasing various cytokines, chemokines, and growth factors that are crucial for cancer cell survival. However, in many cases, their exact cellular targets remain poorly defined. ISG15, a secreted protein and a characteristic gene of cancer-associated myofibroblasts, showed increased expression during the PCT in both scRNA-seq and ST analyses (Figs. S9D and S14A). Among all CAFs across the four disease stages, ISG15 expression was highest in inflammatory CAFs within CCIS and CSCC tissues and further increased in inflammatory CAFs during the PCT (Fig. S14A, B). Therefore, we further investigated the potential cellular interaction between inflammatory CAFs and malignant epithelial cells in the CSCC group by intercellular communication analysis (Fig. S14C). CLDN1 and ISG15 play oncogenic roles in cervical epithelial cells (Fig. 7A–D). Coculture of SiHa cells with human cervical fibroblast cells enhanced cell proliferation and colony formation by activating the FGF1/FGFR1/PI3K/AKT/mTOR signaling pathway (Fig. 7E–H). These data indicate direct contact and tight interaction between inflammatory CAFs and malignant epithelial cells. ISG15 is a ubiquitin-like protein that regulates protein stability by inhibiting ubiquitin-mediated degradation through conjugation with target proteins (ISGylation). We retrieved the structures of ISG15 (PDB: 1Z2M) and FGF1 (PDB: 4YOl) from Protein Data Bank (PDB). Molecular docking analysis performed using an online platform (http://hdock.phys.hust.edu.cn/) predicted that ISG15 could directly bind to FGF1 (Fig. 7I). Surface plasmon resonance (SPR) assay further confirmed this interaction (Fig. 7J). Co-immunoprecipitation (Co-IP) assay validated that ISG15 interacts with FGF1 in cervical cancer cells (Fig. 7K). ISG15 overexpression increased FGF1 protein levels without affecting its mRNA expression, indicating that ISG15 regulates FGF1 degradation at the post-translational level (Fig. 7L, M). We verified that ISG15 overexpression reduced FGF1 degradation in CSCC cells (Fig. 7N). Furthermore, immunoprecipitation assays after ISG15 overexpression revealed that ISG15 binds to FGF1 and protects it from ubiquitin-proteasome-mediated degradation (Fig. 7O). Immunoprecipitation assays in both ISG15 overexpression and knockdown cells indicated that ISG15 competes with ubiquitin for binding to FGF1, thereby enhancing its stability (Fig. 7P–S). We also found that PD161570, an FGFR1 inhibitor, significantly suppressed the proliferation and colony formation ability of CSCC cells (Figs. 7T, U, S14D). We next examined the FGF1-FGFR1-mediated PI3K/AKT signaling pathway, and the results showed that CLDN1 and ISG15 overexpression, as well as PTGDS knockdown, increased the levels of phosphorylated FGFR1, PI3K, AKT, and mTOR (Fig. 7V, W).

#### Targeting the ISG15 and CLDN1-mediated PI3K/AKT pathway as a therapeutic strategy for cervical cancer

**Fig. 8:**
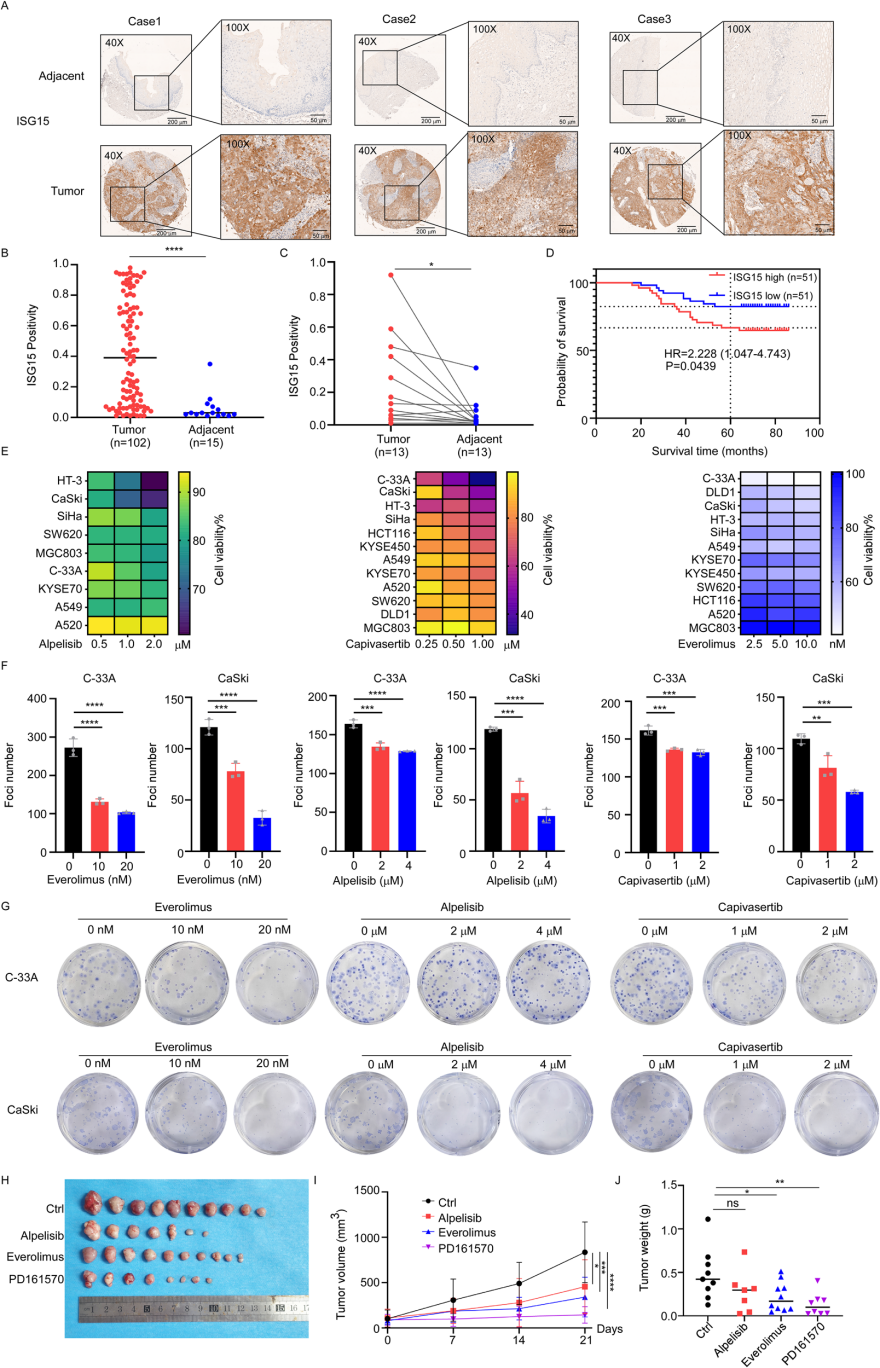
ISG15 expression is correlated with poor prognosis in cervical cancer, FGFR1, PI3K, AKT, and mTOR inhibitors exert antitumor effects in cervical cancer. **A** Immunohistochemical staining of ISG15 in cervical cancer and adjacent tissues with cervical cancer tissue microarray (HUteS120Su01). Representative images at 40× and 100× magnification. Scale bars: 200 µm and 50 µm. **B** Immunohistochemistry evaluation of ISG15 expression in cervical cancer tumor tissues and adjacent tissues. **C** Analysis of ISG15 expression in paired cervical cancer tumor tissues and adjacent tissues. **D** Overall survival of cervical cancer patients stratified by ISG15 expression levels based on tissue array data. **E** Heatmap of cell viability of different cancer cells following treatment with alpelisib, capivasertib, and everolimus. **F**, **G** Colony formation of C-33A and CaSki cells treated with different concentrations of everolimus, alpelisib, and capivasertib. **H-J** Representative images (**H**), tumor volume (**I**), and tumor weight (**J**) from the C-33A cell-derived xenograft model. Mice were divided into four groups: vehicle control, alpelisib (40 mg/kg, oral gavage), everolimus (1 mg/kg, oral gavage), and PD161570 (2 mg/kg, intraperitoneal injection). Tumor volume was measured once a week for 3 weeks. Statistical significance was determined by one-way ANOVA (**F**, **I**, **J**) and Student’s *t*-test (**B**, **C**). The *p-*value < 0.05 was considered statistically significant (*p* < 0.05: *, *p* < 0.01: **, *p* < 0.001: ***, *p* < 0.0001: ****).

To determine the clinical relevance of ISG15 and CLDN1, we examined their expression in cervical cancer using tissue microarrays (Shanghai Outdo Biotech Company, HUteS120Su01 and HUteS30PG01-2, Tables 7, 8). Immunohistochemistry (IHC) analysis revealed significant upregulation of ISG15 and CLDN1 in cervical cancer tissues compared with adjacent normal tissues (Figs. 8A–C, S15A–C). These findings suggest that both genes are strongly associated with tumor progression. Survival analysis further demonstrated that patients with high ISG15 or CLDN1 expression exhibited markedly reduced overall survival, indicating that these genes may serve as prognostic biomarkers for poor outcomes (Figs. 8D, S15D). We validated the expression of CLDN1, ISG15, PTGDS, p-FGFR1, p-AKT, p-mTOR, and Ki67 by IHC in CDX tumor tissues overexpressing CLDN1, ISG15, and PTGDS. The results showed that Ki67, p-FGFR1, p-AKT, and p-mTOR expression were elevated in CLDN1- and ISG15-overexpressed CDX tumor tissues (Fig. S16A, B, D, E), while their levels were reduced in PTGDS-overexpressed CDX tumor tissues (Fig. S16C, F). These results suggest that ISG15 or CLDN1 overexpression, or PTGDS knockdown, enhanced CSCC tumor growth by activating the PI3K/AKT signaling pathway. Currently, there are no clinically available ISG15 inhibitors or PTGDS activators. The U.S. Food and Drug Administration (FDA) has fast-tracked the investigation of the monoclonal antibody ALE.C04 for treating CLDN1-positive head and neck squamous cell carcinoma. To evaluate the sensitivity of CSCC cells to PI3K/AKT signaling pathway inhibitors, we tested several clinically used PI3K, AKT, and mTOR inhibitors in different cancer cells. Clinically approved PI3K inhibitors include alpelisib, duvelisib, idelalisib, and linperlisib, while capivasertib functions as an AKT inhibitor. Duvelisib, idelalisib, and linperlisib are applied for hematologic malignancies. For PIK3CA-mutated, HR-positive, HER2-negative advanced or metastatic breast cancer, alpelisib is administered in combination with fulvestrant. Capivasertib is specifically for locally advanced or metastatic breast cancer. Cervical cancer is a solid epithelial tumor that develops from precancerous lesions. To assess the response of cervical cancer cells to PI3K/AKT pathway inhibition, we used alpelisib (a PI3K inhibitor), capivasertib (an AKT inhibitor), and everolimus (a mTOR inhibitor). The results showed that cervical cancer cells were more sensitive to PI3K/AKT cascade inhibitors than other cancer cells, particularly to capivasertib (Fig. 8E). PI3K/AKT cascade inhibitors also obviously suppressed the colony formation ability of CSCC cells (Fig. 8F, G). These results indicate that targeting PTGDS-, CLDN1-, and ISG15-mediated PI3K/AKT signaling pathway may represent a promising therapeutic strategy for cervical cancer.

**Table 7:** Cohort analysis of cervical cancer patient information (ISG15).

Clinicopathological characteristics		Number (*n* = 102)	Percent (%)	ISG15		*p*-Value
				High (*n* = 51)	Low (*n* = 51)	
Age	Median (range)	47 (30-67)				
Pathology grade	I	1	1.14	0 (0%)	1 (100%)	0.796
	I -II	5	5.74	1 (20%)	4 (80%)	
	II	7	8.04	6 (85.71%)	1 (14.28%)	
	II- III	11	12.64	4 (36.36%)	7 (63.63%)	
	III	63	72.41	32 (50.79%)	31 (49.20%)	
Clinical stage	1	57	55.82	30 (52.63%)	27 (47.36%)	0.455
	2	27	26.47	14 (51.85%)	13 (48.14%)	
	3	17	16.6	6 (35.29%)	11 (64.7%)	
	4	1	0.98	1 (100%)	0 (0%)	
Status	Survival	75	73.52	38 (50.66%)	37 (49.33%)	0.822
	Death	27	26.47	13 (48.14%)	14 (51.85%)

**Table 8:** Cohort analysis of cervical cancer patient information (CLDN1).

Clinicopathological characteristics		Number (*n* = 70)	Percent (%)	CLDN1		*p*-Value
				High (*n* = 35)	Low (*n* = 35)	
Age	Median (range)	47 (31–70)				
Pathology grade	I	1	1.72	0 (0%)	1 (100%)	0.464
	I -II	4	3.77	3 (75%)	1 (25%)	
	II	6	4.88	3 (50%)	3 (50%)	
	II- III	8	6.84	6 (75%)	2 (25%)	
	III	39	35.78	18 (46.15%)	21 (53.85%)	
Clinical stage	1	43	61.43	21 (48.84%)	22 (51.16%)	0.758
	2	17	24.29	8 (47.06%)	9 (52.94%)	
	3	9	12.86	6 (66.67%)	3 (33.33%)	
	4	1	1.43	0 (0%)	1 (100%)	
Status	Survival	49	70	21 (42.86%)	28 (57.14%)	0.68
	Death	21	30	14 (66.67%)	7 (33.33%)	

Importantly, pharmacological inhibition of this pathway using alpelisib, everolimus, or PD161570 led to significant suppression of CSCC tumor volume and tumor weight. PD161570 exhibited a stronger tumor-suppressive effect than alpelisib or everolimus in vivo (Figs. 8H–J, S17A, B). Moreover, PD161570 demonstrated a favorable safety profile and effectively inhibited cervical cancer growth by targeting the FGFR1 signaling pathway (Fig. S17C–E). Furthermore, exploratory analysis of publicly available immunotherapy-treated datasets revealed that elevated ISG15 and CLDN1 expression levels were associated with a poorer response to immune checkpoint blockade therapy (Fig. S17F–M). Collectively, these data suggest that ISG15 and CLDN1 not only drive tumor progression through PI3K/AKT signaling but are also associated with differential immunotherapy response, although validation in dedicated CSCC immunotherapy cohorts is required.

## Discussion

The precancer-to-cancer transition of CSCC represents a crucial stage for CSCC prevention and treatment. Analyzing PCT-associated alterations in CSCC using samples obtained from the same patients enables a more precise understanding of CSCC tumorigenesis and disease progression. However, effective biomarkers and therapeutic targets remain lacking for CSCC diagnosis and treatment identified through longitudinal analyses from the same patient. Here, we utilized scRNA-seq and spatial transcriptomics analyses to identify potential biomarkers and therapeutic targets associated with the PCT of CSCC by examining tissues at different stages from the same CSCC patients. The results revealed significant alterations in subpopulations of fibroblasts, endothelial cells, and B cells. Moreover, we identified a panel of biomarkers and potential therapeutic targets for the diagnosis and treatment of CSCC.

Fibroblasts, a type of stromal cell found in connective tissue, play a crucial role in the TME. Fibroblasts can differentiate into CAFs and acquire an activated state, characterized by increased secretion of ECM components (such as fibronectin, laminin, collagen), production of matrix metalloproteinases, and release of various signaling molecules that promote tumor growth, angiogenesis, immune evasion, metastasis, and drug resistance [18]. Hub genes associated with CAFs may function as important prognostic biomarkers for cervical cancer [19]. Moreover, ECM-CAFs and Myo-CAFs have been reported to function as prognostic indicators of survival in patients with cervical cancer [15, 20, 21]. Endothelial cells, which form the inner lining of blood vessels, are key participants in the TME. Their interactions with tumor cells and other TME elements significantly influence tumor angiogenesis, vascular permeability, immune modulation, formation of pre-metastatic niches, and therapy resistance [22]. B cells, a vital component of the adaptive immune system, play multifaceted roles in the context of cancer, including antibody production, antigen presentation, and immune activation, regulation, and modulation, formation of tertiary lymphoid structures (TLS), regulation of tumor growth and metastasis, underscoring their significance in the TME [22–24].

In this study, we found that the subpopulation of fibroblasts, endothelial cells, and B cells changed significantly during the PCT of CSCC. The GNLY-IR-CAFs and GNLY-IR-ECs increased notably, while IGFBP7-positive progenitor cells decreased significantly during PCT of CSCC. GNLY, a member of the saposin-like protein (SAPLIP) family, possesses cytolytic and proinflammatory properties. GNLY induces the expression of several proinflammatory cytokines, including interleukin (IL)-10, IL-1, IL-6, interferon alpha (IFN-α), macrophage inflammatory protein 1α (MIP1α), monocyte chemoattractant protein 1 (MCP1), MCP3, as well as regulated upon activation T cell expressed and secreted (RANTES) [25]. Numerous studies have demonstrated that GNLY is effective against a broad range of microorganisms, such as parasites, Gram-positive and Gram-negative bacteria, and fungi. However, recent scRNA-seq studies have revealed that GNLY plays critical roles in cancer. GNLY-CD103-cytotoxic T cells with a dysfunctional phenotype have been found to have no association with positive results in colorectal cancer [26]. Moreover, GNLY-positive T cells exhibiting copy number variation levels, stemness, and high proliferative capacity contribute to the malignancy of cutaneous T cell lymphoma [27]. We found that GNLY-IR-CAFs and GNLY-IR-ECs were significantly enriched during PCT of CSCC, suggesting they may drive PCT progression and act as diagnostic markers. Future studies using CAF- or endothelial-specific GNLY knockout models with in vivo immune profiling are needed to determine whether GNLY directly contributes to tumor immunosuppression and CSCC progression and to clarify its mechanistic role in shaping the tumor microenvironment. In addition, we found that IGFBP7 progenitor cells decreased significantly during PCT of CSCC. IGFBP7, a member of the insulin-like growth factor (IGF)-binding protein (IGFBP) family, binds IGFs with high affinity, regulates their availability in the body, and alters their receptor interactions. Low IGFBP7 expression has been reported as an independent prognostic factor in breast cancer [28]. Metformin has been shown to upregulate IGFBP7, thereby inhibiting cervical cancer cell growth [29]. These findings, together with our results, suggest that IGFBP7 may serve as a diagnostic and prognostic marker in cancer. The dynamic changes in fibroblasts, endothelial cells, and B-cell subpopulations highlight the emergence of immune suppression during PCT of CSCC.

Beyond cellular alterations, we also analyzed dysregulated genes during PCT of CSCC and identified CLDN1, ISG15, and PTGDS as key genes exerting significant effects on CSCC progression. To date, few studies have investigated CLDN1, ISG15, and PTGDS in cervical cancer to the best of our knowledge. CLDN1, a member of the claudin protein family, plays a crucial role in tight junctions. ISG15, a ubiquitin-like protein, is essential for the innate immune response against viral infection. Previous studies have reported that CLDN1 and ISG15 may exhibit antitumor effects in CSCC [30, 31]. However, our results demonstrated that CLDN1 and ISG15 are significantly overexpressed in CSCC tissues and serve as oncogenic drivers in CSCC. Cohort analysis in cervical cancer tissue arrays revealed that high ISG15 and CLDN1 expression correlated with poor prognosis, suggesting that these genes may serve as prognostic biomarkers for cervical cancer. PTGDS participates in the contraction and relaxation of smooth muscles and acts as a strong inhibitor of platelet aggregation. Cervical cancer patients with low expression and high-methylation of PTGDS have a worse prognosis than those with high-expression and low-methylation PTGDS [32]. Our results showed that PTGDS has significantly lower expression in CSCC tumor tissues compared with non-tumor tissues, and PTGDS functions as a tumor suppressor in CSCC. Database analyses further confirmed that low PTGDS expression is correlated with poor clinical outcomes in cervical cancer. We also found that the serum PTGDS mRNA levels progressively decreased during the progression of cervical cancer. PTGDS may thus serve as a potential serum biomarker for cervical cancer diagnosis. In clinical practice, combined detection of CLDN1, ISG15, and PTGDS in cervical biopsy specimens may serve as an auxiliary diagnostic indicator to identify patients at higher risk of malignant transformation during PCT and guide personalized monitoring and early intervention.

To elucidate the molecular mechanisms underlying the PCT mediated by CLDN1, ISG15, and PTGDS, we conducted transcriptomic sequencing, along with a series of in vitro and in vivo experiments. We found that CLDN1, ISG15, and PTGDS drive CSCC progression during the PCT through the FGF1-FGFR1-mediated PI3K/AKT signaling pathway. The PI3K/AKT signaling pathway is a well-characterized therapeutic axis in cancer, for which specific inhibitors are already available in clinical settings. We verified that cervical cancer cells were more sensitive to PI3K/AKT inhibitors than several other cancer types, indicating that CLDN1, ISG15, and PTGDS-mediated FGF1/FGFR1/PI3K/AKT signaling pathway may represent a potential therapeutic target for CSCC. High ISG15 and CLDN1 expression, along with low PTGDS expression, promoted cervical cancer growth in vivo by activating the PI3K/AKT signaling pathway. PI3K/AKT/mTOR and FGFR1 inhibitors effectively suppressed CSCC tumor growth in vivo. Notably, FGFR1 inhibitors demonstrated superior antitumor efficacy compared to PI3K or mTOR inhibitors, highlighting their greater potential as therapeutic targets for cervical cancer. These findings suggest that FGFR1/PI3K/AKT/mTOR inhibitors may represent potential therapeutic candidates for cervical cancer, and clinical translation of these agents may significantly improve patient outcomes. Although our conclusions were validated using tissue samples from patients at various stages and time points, additional patient cohorts are needed to further validate the clinical relevance of these biomarkers. Collectively, we demonstrated that the CLDN1-, PTGDS-, and ISG15-mediated FGF1/FGFR1/PI3K/AKT signaling pathway represents a potential therapeutic target for CSCC.

Cancer-associated fibroblasts, as a vital part of the tumor microenvironment, promote malignant growth through multiple mechanisms. However, the heterogeneity of CAFs has made it difficult to investigate their interactions with tumor cells. Here, we characterized CAFs and confirmed their presence in human CSCC using multiplex immunofluorescent labeling. Our findings revealed that CAFs highly express genes associated with pro-tumorigenic pathways. Intercellular communication analysis suggested that CAFs promote CSCC progression primarily through activation of FGF1 signaling. Specifically, ISG15, synthesized by CAFs, stabilizes FGF1, leading to activation of FGFR1 and subsequent stimulation of the PI3K/AKT signaling cascade (Fig. 7W). PD161570 exhibited strong potential as a targeted therapeutic agent in cervical cancer. Collectively, these findings provide mechanistic insight and highlight potential targets for early intervention in CSCC. These findings support further exploration of CLDN1-targeting antibodies, ISG15 inhibitors, PTGDS activators, and FGFR1/PI3K/AKT/mTOR inhibitors in translational settings. In summary, this study demonstrates that the GNLY-IR-CAFs, GNLY-IR-ECs, as well as IGFBP7-progenitor cells are dysregulated during PCT of CSCC. CLDN1, PTGDS, and ISG15 were identified as novel biomarkers and potential therapeutic targets for CSCC. We validated the diagnostic potential, biological functions, and underlying mechanisms of CLDN1, PTGDS, and ISG15 in CSCC tissues and cells. More importantly, we evaluated the therapeutic potential of CLDN1, PTGDS, and ISG15, as well as their involvement in the FGF1/FGFR1/PI3K/AKT signaling pathway.

Validation in both HPV-positive (CaSki, SiHa) and HPV-negative (C-33A) cell lines further supported the role of CLDN1/ISG15/PTGDS and the ISG15-FGF1 axis. Results in C-33A indicate that these genes, especially ISG15-mediated FGF1 stabilization, contribute to cervical cancer progression independent of HPV infection. These findings suggest that the ISG15-FGF1 axis represents a regulatory mechanism that is not restricted to HPV-driven tumorigenesis. Therefore, targeting the ISG15-FGF1 signaling pathway may provide a potential therapeutic strategy for HPV-negative CSCC, for which targeted treatment options remain limited.

According to single-cell RNA sequencing, alterations in fibroblast, endothelial cell, and B cell clusters indicated an immunosuppressive state during the precancer-to-cancer transition of CSCC. To further explore the potential clinical relevance of CLDN1 and ISG15 in the context of immunotherapy, we performed an exploratory analysis using publicly available immunotherapy-treated datasets. We observed that higher CLDN1 and ISG15 expression levels were associated with reduced response to immune checkpoint blockade therapy. However, it should be emphasized that this analysis was based on retrospective public datasets rather than a dedicated CSCC immunotherapy-treated cohort and therefore indicates association rather than definitive predictive value. Further validation in well-defined CSCC cohorts receiving immune checkpoint inhibitors is required. These findings suggest a potential link between CLDN1/ISG15 expression and immunotherapy response, which warrants future investigation.

Compared with previous single-cell or spatial studies, our work uniquely analyzes continuous lesions from the same patients and identifies PCT-associated GNLY-fibroblasts/-endothelial cells, functionally validates a PCT-related gene panel, and reveals a CAFs-derived ISG15-FGF1 axis driving CSCC progression. These findings provide mechanistic insights beyond prior descriptive studies. A limitation of this study is the relatively small number of patients included in the single-cell analysis. This is mainly due to the difficulty of obtaining multi-stage tissue samples from the same patient, which requires strict clinical criteria and coordinated longitudinal collection. Although the sample size is limited, the multi-stage specimens from each individual allow us to capture dynamic cellular changes within patients. To compensate for this limitation, we validated the major findings in a larger independent patient cohort using downstream assays. The consistency of these results supports the robustness and generalizability of our conclusions despite the limited initial sample size. In addition, disease-free survival (DFS) follow-up data were unavailable in the current cohort, preventing assessment of the predictive value of ISG15 and CLDN1 for CSCC recurrence. Larger cohorts with long-term follow-up are needed for validation.

## Material and methods

### Patients and sample collection

Three female patients with a pathological diagnosis of CSCC (HPV16-positive, HPV58-positive) were enrolled from the Affiliated Cancer Hospital of Zhengzhou University, Henan Cancer Hospital, Zhengzhou, China (Table 1). Fresh normal, premalignant, and malignant tissues were surgically resected from the enrolled patients. None of the patients received chemotherapy or radiotherapy before the collection of tissue samples. Following the tissue resection, the tissues for scRNA-seq were stored in tissue protection fluid, and tissues for spatial transcriptomics were subjected to optimal cutting temperature (OCT) embedding after washing and removing unqualified tissues immediately.

### 10x single-cell sequence materials and methods

#### Tissue dissociation and preparation of single-cell suspensions

The obtained NCU, high-grade intraepithelial neoplasia of the cervix, cervical carcinoma in situ, and invasive cervical carcinoma tissue samples were washed three times in pre-cooled PBS on ice. They were then transferred to sterile, RNase-free dishes and dissected into small fragments with sterile surgical scissors. To remove non-target tissues such as blood stains, fatty layers, and connective tissues, samples were further washed with PBS. The samples were incubated at 37 °C in a thermostatic water bath using the hydrolysate kit (Human Tumor Dissociation Kit, Miltenyi Biotec, Cat. No. 130-095-929). Then the cells were filtered through a 40 µm cell sieve and centrifuged at 300 × *g* for 5 minutes at 4 °C. The supernatant was discarded, and the cells were resuspended in 3 mL of pre-cooled erythrocyte lysis buffer (Solarbio), mixed gently by pipetting up and down. Once the cell concentration reached 700–1200 cells/µL, with viability above 90% and aggregation below 15%, the samples were placed on ice, and the 10x Genomics single-cell transcriptome chip experiment was initiated within 30 minutes.

#### Single-cell RNA sequencing library preparation and sequencing

ScRNA-seq libraries were generated using Chromium Next GEM Single Cell 3’ Reagent Kits v3.1 on the Chromium Controller (10x Genomics). Cells were suspended in PBS with 0.04% BSA and loaded onto a Chromium Next GEM Chip G. According to the manufacturer’s protocol, the Chromium Controller was used to generate single-cell gel beads-in-emulsion (GEMs). Each cell was lysed within its respective GEM, enabling barcoding of the released RNA during reverse transcription. Barcoded, full-length cDNA was synthesized, and libraries were constructed according to the manufacturer’s protocol. Library quality was assessed by Qubit 4.0 and Agilent 2100. Sequencing was conducted on an Illumina NovaSeq 6000 platform with 150 bp paired-end reads (PE150) at a depth of ≥50,000 reads per cell, performed by Biomarker Technologies Corporation (Beijing, China).

#### Processing raw data and assay from scRNA-seq of 10x Genomics

Alignment was performed using Cell Ranger 6.0.0, which utilizes the STAR aligner, against a customized human reference genome based on GRCh38. A total of 39,120 cells passed initial quality control. Low-quality cells were excluded by retaining only those with >500 genes and <20% mitochondrial gene content. Genes detected with at least one feature count in more than three cells were retained for subsequent analysis. Finally, we obtained 31,812 high-quality cells for further analysis. Gene expression counts were quantified using unique molecular identifiers (UMIs) for each cell barcode-gene pair. After read alignment, Cell Ranger 6.0.0 was applied to filter barcodes and retain only those corresponding to real cells. These curated barcodes were then used for subsequent analyses, including clustering, cell type annotation, and differential expression testing with Seurat (version 4.0.1).

#### Dimensionality reduction

Dimensionality reduction was performed via principal component analysis (PCA) on the combined samples for each tissue to enable unsupervised clustering and cell type resolution. Subsequently, we used Seurat to reduce the dimensionality of 31,812 cells and applied t-SNE for two-dimensional visualization. Gene expression values were computed using the NormalizeData function in Seurat with the LogNormalize method. Next, PCA was performed on the normalized expression value, and the top 10 principal components (PCs) were selected for both clustering and t-SNE projection. Cell clusters were identified using a graph-based clustering approach that constructs a weighted Shared Nearest Neighbor (SNN) graph. Cluster-specific marker genes were detected using Seurat’s FindAllMarkers function with the bimod (likelihood ratio test) under default parameters. Markers were filtered based on fold change >1.5 and FDR <0.1, and the top 10 genes per cluster were selected as representative marker genes.

#### Clustering and cell type identification

We identified clusters by applying Louvain community detection to a nearest neighbor graph generated in PCA space. To determine cell types, we trained a neural network-based model that classifies scRNA-seq mRNA expression profiles into corresponding cell ontology categories. Cell types were first automatically annotated using SingleR based on seven reference datasets. Manual curation was then performed to add cell state-level annotations to SingleR, enabling more granular classification than provided by default ontology classes. Following initial annotation, the identified cell types were further validated and refined using the CellMarker database (http://bio-bigdata.hrbmu.edu.cn/CellMarker/).

#### Gene ontology enrichment analysis, cell cycle scoring

Gene set enrichment analysis was first performed using Enricher against the Gene Ontology Biological Process 2018 gene sets. Separately, Hallmark gene sets from MSigDB were used to compute enrichment scores using Fisher’s exact test. Both enrichment analyses were corrected for multiple hypothesis testing using the Benjamini-Hochberg method. To assess cell cycle activity, we calculated expression scores for gene signatures corresponding to the S and G2/M phases, as previously described and implemented in Seurat version 4.0.1.

#### Trajectory Inference

Pseudotime trajectory analysis was carried out using Monocle 2 (version 2.18.0, https://github.com/cole-trapnell-lab/monocle-release), which employs reversed graph embedding (RGE), a machine learning approach, to reconstruct intricate single-cell developmental paths. This tool identifies genes associated with the biological process without requiring prior assumptions about the number of cell fates, branching events, or experimental design. Compared with both its earlier release and several newer algorithms, Monocle 2 achieves more precise and stable trajectory reconstructions. The workflow of Monocle 2 consists of three principal stages. First, genes with “interesting” (not just noisy) expression variation were selected to construct the data. Next, the dataset was reduced in dimensionality using these genes. Finally, pseudotime analysis is performed by embedding cells into a low-dimensional space to infer differentiation paths.

### 10x Visium spatial transcriptomics materials and methods

#### Slide preparation

Spatial transcriptomics slides were designed with four identical 6.5 × 6.5 mm capture zones, each comprising 5000 spots embedded with barcoded primers (10x Genomics). These primers, anchored by their 5’ ends, include a partial read 1 Illumina handle, a 20-bp poly(T)VN sequence, a unique molecular identifier (UMI), a T7 promoter region, and a cleavage site. Each spot, measuring 55 μm in diameter, is positioned in a uniform centered hexagonal pattern, surrounded by six neighboring spots with 110 μm center-to-center spacing. Frozen tissue was cut into 10 μm sections using a precooled cryostat, carefully mounted onto chilled Visium Tissue Optimization Slides (3000394, 10x Genomics) and Visium Spatial Gene Expression Slides (2000233, 10x Genomics), and stored at -80 °C until further processing.

#### Tissue optimization

Following recommended guidelines, the spatial transcriptomics protocol was tailored for the tissue samples. Briefly, the staining process was modified by omitting isopropanol, shortening the exposure to hematoxylin and bluing buffer, and raising the eosin concentration. Furthermore, the incubation time for tissue permeabilization was optimized. Simultaneously, the established one-step protocol for tissue removal was revised by increasing the ratio of proteinase K to PKD buffer. Following the establishment of these optimized conditions, three cryosections per patient, each 10 μm thick, were sectioned onto spatial slides and processed without delay.

#### Fixation, staining, and imaging

The sectioned slides were incubated at 37 °C for 1 minute, followed by fixation in 3.7–3.8% formaldehyde (Sigma-Aldrich) prepared in PBS (Medicago) for 30 minutes. Slides were subsequently washed with PBS. For histological staining, sections were immersed in Mayer’s hematoxylin (Dako, Agilent, Santa Clara, CA) for 4 minutes, treated with bluing buffer (Dako) for 30 seconds, and counterstained with Eosin (Sigma-Aldrich) diluted 1:5 in Tris-base buffer (0.45 M Tris, 0.5 M acetic acid, pH 6.0) for another 30 seconds. RNase- and DNase-free water was used to wash the slides after each staining step. Following air drying, slides were mounted using 85% glycerol (Merck Millipore, Burlington, MA) and coverslips (Menzel-Glaser). Bright-field (BF) images were acquired at 20× magnification by the Metafer Slide Scanning platform (MetaSystems), and raw images were stitched using VSlide software (MetaSystems). After imaging, coverslips and glycerol were removed by immersing the slides in RNase and DNase-free water. The slides were then placed into slide cassettes, allowing tissue sections to be isolated into separate reaction chambers (hereafter wells). For pre-permeabilization, sections were incubated at 37 °C for 20 min with 0.5 U/mL collagenase (ThermoFisher) and 0.2 mg/mL BSA (NEB, Ipswich, MA) in HBSS buffer (ThermoFisher). Wells were washed with 0.1 × SSC (Sigma-Aldrich), after which permeabilization was conducted at 37 °C for 7 minutes in 0.1% pepsin (Sigma-Aldrich) dissolved in 0.1 M HCl (Sigma-Aldrich). After incubation, the pepsin solution was removed, and the wells were subsequently washed using 0.1 × SSC.

#### Spatial library preparation and sequencing

Following reverse transcription, the wells were washed with 0.1 × SSC and incubated at 56 °C for 1.5 hours with intermittent shaking in a tissue removal solution composed of Proteinase K (QIAGEN) and PKD buffer (QIAGEN, pH 7.5) mixed at 1:1 ratio. Spatially barcoded cDNA was then released enzymatically, following established protocols. The supernatants were collected and transferred into 96-well plates for library preparation using the automated MBS 8000 system. Briefly, second-strand cDNA synthesis was followed by in vitro transcription, adaptor ligation, and a second reverse transcription step. Indexing PCR was then performed to add sequencing handles and sample barcodes. Final libraries were purified and quantified as described earlier.

#### Spot visualization and image alignment

Primer spots were stained through hybridization with fluorescent probes and imaged via the Metafer Slide Scanning platform. The corresponding spot image and the earlier acquired BF tissue image of the same area were loaded into the web-based ST Spot Detector tool. After image alignment between the two images, tissue-covered spots were identified using the platform’s built-in tissue recognition algorithm.

#### Upstream analysis

The prepared libraries were diluted to 4 nM and subjected to paired-end sequencing on the Illumina NovaSeq 6000 platform. Upstream data processing was performed using Space Ranger (v1.2.2), and reads were aligned to the GRCh38_release95 human reference genome.

#### Downstream analysis

Briefly, data analysis was conducted by steps of normalization, clustering, and screening marker genes through the R package Seurat (v4.0.1), followed by marker gene annotation, functional enrichment analysis using the R package clusterProfiler (v3.18.1), and protein-protein interaction network construction by the STRING database and R packages igraph and ggraph. Lastly, we performed transcription factor binding site prediction through the TFBStools R package, using AnimalTFDB (v3.0, http://bioinfo.life.hust.edu.cn/AnimalTFDB/) as the transcription factor motif reference database. We used default parameters to filter data for analysis. Visualization was performed mainly through the R package ggplot2.

#### Plasmid construction

Eukaryotic expression plasmids, including pcDNA3.1-Flag-C CLDN1, pcDNA3.1-Flag-C ISG15, pcDNA3.1-Flag-C PTGDS, pcDNA3.1-Flag-C GNLY, pcDNA3.1-MYC-C, pcDNA3.1-MYC-C FGF1, pcDNA3.1-HA-ubiquitin, were purchased from Youbao Biotechnology Company, Changsha, China.

#### RNA interference

PTGDS shRNAs were purchased from GenePharma. The shRNAs were as follows: shNC-GFP-Puro (GTTCTCCGAACGTGTCACGT), shPTGDS#1 (ACTCATCACACACTGTGGATG), shPTGDS#2 (CCAACTTCCAGCAGGACAAGT), and lentiviral vectors containing shRNAs targeting ISG15 were ligated to the pLKO.1 vector. The shRNA primers of ISG15 knockdown were as follows: shISG15#1-F: CCGGCTGAGCATCCTGGT GAGGAATCTCGAGATTCCTCACCAGGATGCTCAGTTTTTG, shISG15#1-R: AATTCAAAAACTGAGCATCCTGGTGAGGAATCTCGAGATTCCTCACCAGGATGCTCAG. shRNA plasmids, along with psPAX2 and pMD2G packaging plasmids, were transfected into HEK293T cells. After 48 h of transfection, lentiviral particles were collected and filtered through 0.22 μm filters. C-33A and CaSki cells were then infected with the virus supplemented with polybrene (8 μg/mL, Millipore, Cat#TR-1003-G) for 24 hours. Cells were selected using puromycin (5 μg/mL). Small interfering RNA (siRNA) targeting ISG15, CLDN1, and PTGDS was purchased from GenePharma company. Each siRNA (75 nmol/L) was transfected into the cells using JetPRIME. The siRNA had the following sequences: siNC (S: UUCUCCGAACGUGUCACGUTT), siISG15#1 (S: GCAUCCUGGUGAGGAAUAATT), siISG15#2 (S: GCACCGUGUUCAUGAAUCUTT), siCLDN1#1 (S: CUGGGAGUGAUAGCAAUCUTT), siCLDN1#2 (S: GGUAUGGCAAUAGAAUCGUTT), siPTGDS#1 (S: GUUGUCCAUGUGCAAGUCUTT), siPTGDS#2 (S: AGACCGACUACGACCAGUATT).

#### Cell proliferation assay and crystal violet staining assay

Cells were seeded at a density of 2000 cells per well in 96-well plates for the cell proliferation assay. The next day, cells were treated with 20 μL of MTT solution (5 mg/mL) for 2 h. 200 μL DMSO was used to dissolve the MTT formazan. Optical density (OD) values at 490 nm or 570 nm were measured to evaluate cell proliferation ability. Three parallel wells were included for each group. In the crystal violet staining assay, 600 cells were seeded per well in a 6-well plate and cultured in a CO_2_ incubator. After 2 to 3 weeks, cells were stained with a 0.3% crystal violet solution (dissolved in methanol) for 30 minutes at room temperature and washed three times with distilled water. Three parallel wells were included for each group. The plates were photographed, and colony numbers were subsequently counted.

#### Quantitative real-time PCR analysis

Total RNA from blood was extracted with the Blood RNA Extraction Kit (Haigene, B0901), followed by reverse transcription with the Prime RT Reagent Kit (Vazyme, Cat#R323). mRNA levels were quantified using SYBR qPCR Master Mix (Vazyme, Cat#Q711) by real-time PCR system. Three parallel wells were included for each group. mRNA levels were normalized to GAPDH. The qRT-PCR primer sequences are listed below. SERPINB3(SCCA)-F, GAGAACACCACAGGAAAAGCTGC, SERPINB3(SCCA)-R, TCTCCGAAGAGCTTGTTGGCGA; CLDN1-F, GTCTTTGACTCCTTGCTGAATCTG,CLDN1-R, CACCTCATCGTCTTCCAAGCAC; PTGDS-F, AGCCCAACTTCCAGCAGGACAA,PTGDS-R, CAGACTTGCACATGGACAACGC; ISG15-F, CTCTGAGCATCCTGGTGAGGAA, ISG15-R, AAGGTCAGCCAGAACAGGTCGT; IGKC-F, CACAAAGTCTACGCCTGCGA, IGKC-R, GGGGCACTTCTCCCTCTAAC; FGF1-F, ATGGCACAGTGGATGGGACAAG, FGF1-R, TAAAAGCCCGTCGGTGTCCATG.

#### Cell culture

Human cervical epithelial cells (CEC) were purchased from ZQXZBIO (Shanghai, China). Human cervical epithelial cells were cultured in complete culture medium for human cervical epithelial (PCM-H-070, ZQXZBIO). Human cervical fibroblasts were purchased from iCell (Shanghai, China) and cultured in specialized medium (iCell-f013-002h, iCell). The human cervical cancer cell lines CaSki, SiHa, HT-3, and C-33A were purchased from ZQXZBIO (Shanghai, China). CaSki cells were cultured in RPMI 1640 medium. SiHa and C-33A cells were cultured in modified Eagle’s medium (MEM). HEK293 and HEK293T cells were obtained from ATCC and cultured in DMEM (high glucose) (Biological Industries). HT-3 cells were cultured in Mycoy’s 5 A medium. The human ESCC cell lines (KYSE70 and KYSE450), maintained in RPMI-1640 medium, were preserved by the Department of Pathophysiology at the School of Basic Medical Sciences, Zhengzhou University. Human lung cancer cells (A520, A549), human colon cancer cells (HCT-116, DLD1, SW620) were obtained from ATCC and cultured in RPMI-1640 medium. RPMI-1640 medium, MEM medium, DMEM medium, and MyCoy’s 5A medium were supplemented with 10% FBS (VivaCell, Cat#2302008). Authentication of these cell lines was performed using STR analysis. All cell lines were cultured at 37 °C in a humidified incubator with 5% CO_2_.

#### Reagents and antibodies

The reagents we used in this study were as follows: PI3Kα inhibitor alpelisib (HY-15244, MedChemExpress), pan-AKT inhibitor capivasertib (HY-15431, MedChemExpress), mTOR1 inhibitor everolimus (HY-10218, MedChemExpress), cycloheximide (HY-12320, MedChemExpress), FGF1 receptor inhibitor PD161570 (HY-100434, MedChemExpress), proteasome and calpain inhibitor MG132 (HY-13259, MedChemExpress), autophagy inhibitor 3-Methyladenine (HY-19312, MedChemExpress), Recombinant human ISG15 protein (I245-31H, Sino Biological), Recombinant human FGF1 protein (ab91374, abcam). The following antibodies were used in our study: anti-ISG15 (ab285367, abcam), anti-PTGDS (ab182141, abcam), anti-CLDN1(ab211737, abcam), anti-GAPDH (#HRP-60004, Proteintech), anti-IGFBP7 (ab171085, abcam), Anti-IGKC Mouse monoclonal antibody (HA601099, Huabio), Anti-GNLY/Granulysin (ab241333, abcam), Anti-Collagen III (ab6310, abcam), Anti-Von Willebrand Factor (ab201336, abcam), Anti-FGF1 (ab207321, abcam), Anti-FGFR1 (phospho Y654, ab59194, abcam), Anti-FGF Receptor 1 (9740S, CST), Anti-p-AKT (Thr308) (13038, CST), Anti-p-AKT (Ser473) (4060, CST), Anti-AKT (4691, CST), Anti-p-mTOR (Ser2448) (5536, CST), Anti-mTOR (2983, CST), Anti-PI3K p85 (4257, CST), Anti-PI3K p85 Y458/p55 Y199 (17366S, CST), Anti-Ki67 (ab16667, abcam), Anti-CDKN2A/p16INK4a (ab241543, abcam), Anti-ubiquitin (10201-2-AP, Proteintech).

#### Computer docking

The structures of ISG15 (PDB ID: 1Z2M), FGF1 (PDB ID: 4YOl) were obtained from the Protein Data Bank (PDB). Protein-protein interactions were analyzed using the standard procedures on the website (http://hdock.phys.hust.edu.cn/).

#### Western blot analysis

Cell pellets were resuspended using RIPA lysis buffer (Solarbio, R0020) containing 1% protease and phosphatase inhibitor Cocktail (NCM Biotech, Cat# P002). Cells were incubated on ice for 30 minutes to allow complete lysis. After centrifugation at 14,000 rpm for 15 minutes at 4 °C, the supernatant was collected as whole cell extracts. Protein concentration was measured using the BCA Quantification Kit (Solarbio, Beijing, China, Cat#PC0020). SDS-PAGE was used to separate proteins, which were subsequently transferred onto polyvinylidene difluoride (PVDF) membranes. After blocking with 5% skimmed milk for 1 hour, the membranes were incubated with the primary antibody overnight at 4 °C. They were then exposed to a horseradish peroxidase-linked secondary antibody for 1.5 hours at room temperature (RT). Target protein bands were detected via enhanced chemiluminescence (ECL) and imaged using the Amersham Imager 800 (GE Healthcare Life Science, Pittsburgh, PA, USA).

#### Multiplex immunofluorescent labeling

Tissue sections from patients were collected on formalin-fixed paraffin-embedded (FFPE) slides (4 µm), then deparaffinized, rehydrated, and treated with citrate buffer for 90 s. The sections were incubated with IGFBP7 (1:100), GNLY (1:250), IGKC (1:50), VWF (1:100), COL3A1 (1:100), CLDN1(1:50), ISG15 (1:100), and PTGDS (1:200) antibodies (in solution containing 2% BSA, 0.3% Triton X-100, 1% Normal Goat serum, 0.1% NaN3) at 4 °C overnight. The tissue sections were washed with PBST and incubated with secondary antibody, Goat anti-Rabbit IgG (H + L) Cross-Adsorbed Secondary Antibody, Cyanine3 (1:1000; Invitrogen), Goat anti-Mouse IgG (H + L) Cross-Adsorbed Secondary Antibody, FITC (F2761, Invitrogen), Goat anti-Mouse IgG (H + L) Cross-Adsorbed Secondary Antibody, Alexa Fluor™ 568 (A11004, Invitrogen), Goat anti-Rabbit IgG (H + L) Highly Cross-Adsorbed Secondary Antibody, Alexa Fluor™ 488 (A11034, Invitrogen) for 2 h at room temperature, after which tissue sections were stained with DAPI solution (C0060, Solarbio) for 20 min. Images were acquired using the Akoya Biosciences imaging system. Data was quantified from three independent fields captured for each sample.

#### Immunoprecipitation assay (IP)

Equivalent cell lysates were incubated with normal IgG antibody and specific antibodies. The mixture was rotated at 4 °C overnight. 50 µL Protein A/G magnetic beads were added and rotated at 4 °C for 4 h. The beads were washed three times with IP wash buffer. 1× Loading buffer was added to each tube, and samples were heated at 95 °C for 4 minutes. Blank lysate was used as an input control. A Western blot was then performed.

#### Cell coculture assay

Human cervical fibroblasts were seeded at 6 × 10^5^ cells/mL in 10 cm dishes and cultured overnight. The cells were cultured in a CO_2_ incubator for 24 h in serum-free medium. After 48 h, the conditional medium (CM) was collected and centrifuged at 5000 rpm for 10 minutes to remove cell debris. The supernatant was filtered through 0.22 μm filters. Cervical cancer cells were seeded in 96-well plates, cultured for 24 h, and treated with mixed medium (CM: culture medium, 1:1). Cell proliferation was assessed by MTT assay at 0, 24, 48, and 72 h.

#### Immunohistochemistry

Human tissue sections, with a thickness of 4 µm, were collected on formalin-fixed paraffin-embedded (FFPE) slides. The human NCU, CIN, and CSCC tissue sections were deparaffinized and rehydrated in citrate buffer (10 mmol/L, pH 6.0) for 90 s. Slides were incubated with 3% H_2_O_2_ for 10 min to block the endogenous peroxidase activity and blocked the slides with 10% goat serum for 1 h at RT. Then, the slices were treated with specific primary antibodies (ISG15, 1:500; CLDN1, 1:250; PTGDS, 1:200; KI67, 1:200; CDKN2A/p16INK4a, 1:640; p-AKT S473, 1:100; p-FGFR1 Y654, 1:100) at 4 °C overnight. The tissue sections were then treated with a secondary antibody, followed by staining with 3,3’-diaminobenzidine (DAB). Image-Pro Plus 6.0 software was used to calculate the integrated optical density (IOD), and the corresponding log_10_(IOD) values are presented in the figures. Data was quantified from three independent fields captured for each sample.

#### Cell-derived xenograft mouse models

Animal experiments were conducted with approval from the Ethics Committee of China-US (Henan) Hormel Cancer Institute (Zhengzhou, Henan, China). Female mice (6-8 weeks old) (Vital River Labs, Beijing, China) were randomly assigned to different groups by body weight. Each mouse was subcutaneously injected with 5 × 10^6^ cells from either the CaSki, C-33A, or SiHa cell lines. Mouse weight and tumor volume were measured weekly. Tumor volume was calculated using the formula: (length) × (width) × (height). In the inhibitor-treated CDX model, mice were allocated into four treatment groups with comparable baseline tumor volumes following the establishment of xenografts. Each group received a distinct intervention: vehicle control, alpelisib administered by oral gavage (40 mg/kg/day), everolimus administered by oral gavage (1 mg/kg/day), and PD161570 (2 mg/kg/day) administered intraperitoneally. The mice were sacrificed, and tumor tissues were extracted for further analysis.

#### Surface plasmon resonance assay

The CM5 sensor chip was installed into the instrument according to the manufacturer’s instructions. The ISG15 protein was immobilized on the chip surface, and different concentrations of FGF1 were sequentially applied to the surface, as described in the Biacore Sensor Surface Handbook, to investigate the binding interactions between ISG15 and FGF1. The data obtained were analyzed using GraphPad Prism software.

#### Hematoxylin and Eosin staining (H&E)

Tissue sections were deparaffinized in xylene and rehydrated through a graded ethanol series (100%, 95%, 75%, and distilled water). The slides were then stained with Harris hematoxylin for 5 minutes, differentiated in 1% acid alcohol, and blued in 0.2% ammonia water. Sections were subsequently stained with 1% eosin Y for 1–2 minutes, dehydrated through ethanol, cleared in xylene, and mounted with neutral resin. The stained slides were observed under a light microscope.

#### Immunotherapy response data acquisition

Treatment response data were obtained from the KM Plotter online platform (https://kmplot.com/analysis/). The database includes transcriptomic and clinical response data from GEO cohorts of solid tumors treated with immune checkpoint inhibitors (anti-PD-1, anti-PD-L1, and anti-CTLA-4). Patients were classified as responders or non-responders according to the KM Plotter annotation. This analysis was conducted for exploratory purposes.

#### Statistical analysis

All statistical analysis in this study was done with GraphPad Prism 8.0 or SPSS 22.0. To compare significant differences, the Student’s t-test, one-way analysis of variance (ANOVA), Wilcoxon rank-sum test were utilized. Each experiment was given a significance level of *P < 0.05, **P < 0.01, ***P < 0.001, ****P < 0.0001.

## Supplementary information

SUPPLEMENTARY MATERIAL

Original Western blot

## Data Availability

The datasets supporting the conclusions of this article are included within the article and its additional files. The raw RNA-seq data generated in this study have been deposited in the Sequence Read Archive (SRA) under BioProject ID PRJNA1376270. The raw sequence data are available under restricted access for research purposes only. Access can be obtained through the SRA data access committee (DAC). According to the guidelines of SRA, all non-profit researchers are allowed access to the data, and the Principal Investigator of any research group is allowed to apply for controlled-access data. Users can register on the SRA website (https://www.ncbi.nlm.nih.gov/sra). The approximate response time for accession requests is about 3 days. The access authority can be granted for Research Use Only. Users may also contact the corresponding author directly. Once access has been approved, the data will be available for download for 2 months. The raw scRNA-seq and spatial transcriptomics data generated in this study have been deposited in the GSA-Human database under accession code HRA015365. The raw sequence data are available under restricted access for research purposes only, access can be obtained by the DAC (Data Access Committees) of the GSA-human database. According to the guidelines of GSA-human, all non-profit researchers are allowed access to the data, and the Principal Investigator of any research group is allowed to apply for Controlled-access of the data. The user can register in the GSA database (https://ngdc.cncb.ac.cn/gsa-human/) and request the data. The approximate response time for accession requests is about 3 days. The access authority can be obtained for Research Use Only. The user can also contact the corresponding author directly. Once access has been approved, the data will be available for download for 2 months. The remaining data are available within the article, supplementary information, or source data files. Source data are provided with this paper.
