# Supplementary material for: Integrated multi-omics profiling of treatment-naive cervix uteri premalignant lesions and cervical squamous cell carcinoma reveals ecosystem and drivers underlying cervical cancer progression
Source: Cell Death Dis. 2026 Jun 9;17(1):722. doi: 10.1038/s41419-026-08960-2 (PMC13469616; doi:10.1038/s41419-026-08960-2)

Original Western blot

Figure 6

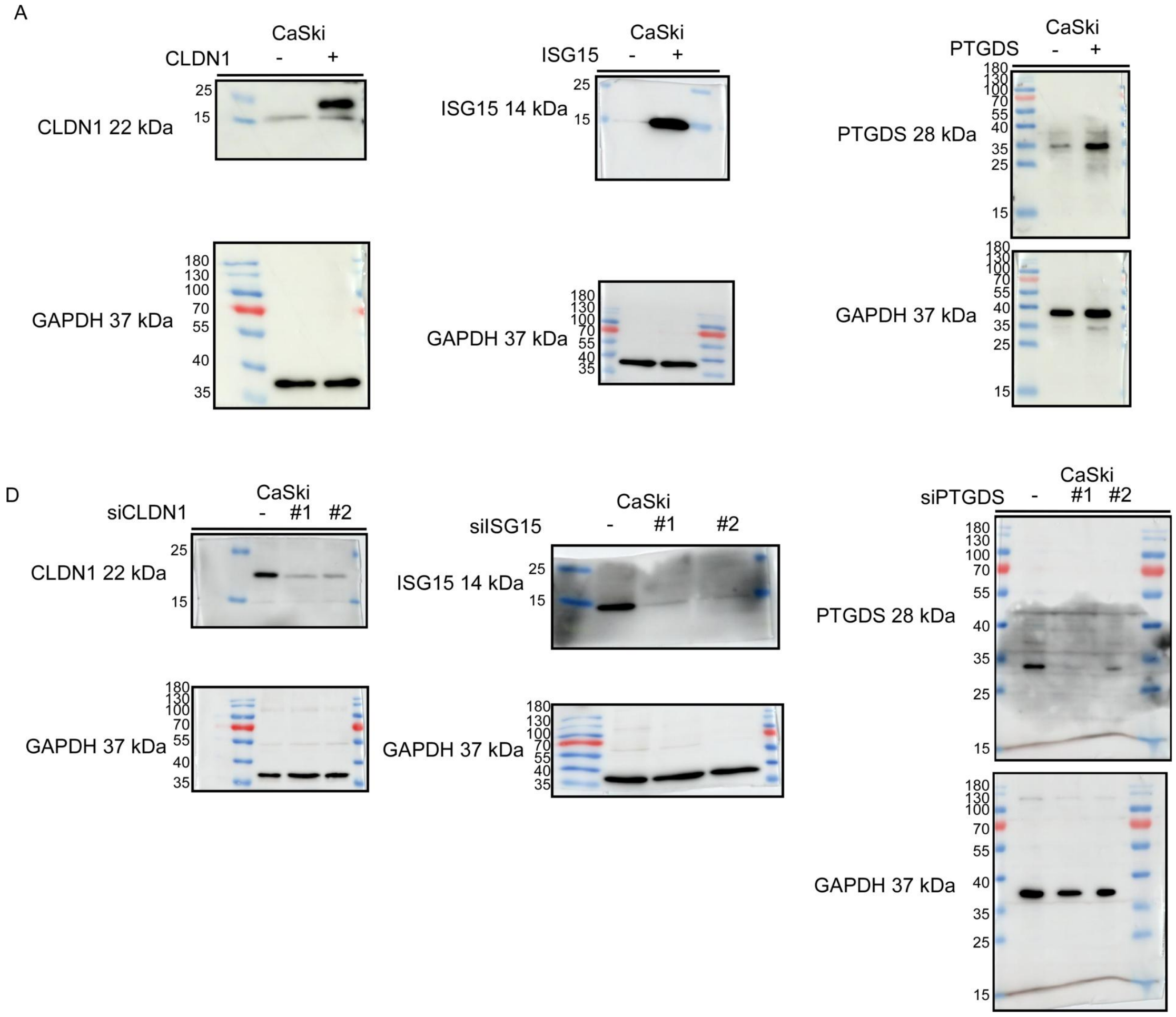

Figure 7

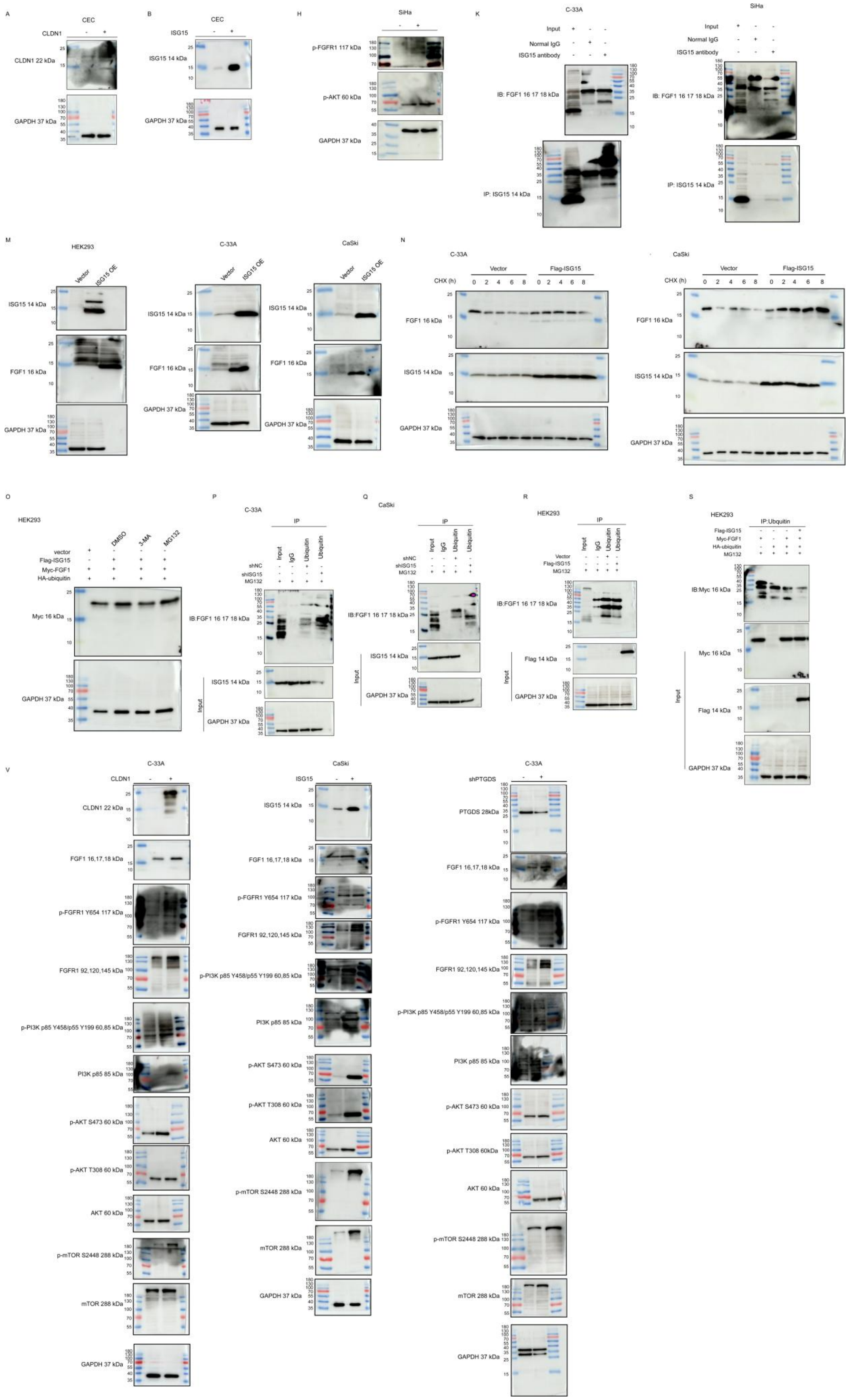
A
CEC
CLDN1 22 kDa
GAPDH 37 kDa
B
ISG15 14 kDa
H
SiHa
p-FGFR1 117 kDa
p-AKT 60 kDa
K
C-33A
Input
Normal IgG
ISG15 antibody
IB: FGF1 16 17 18 kDa
IP: ISG15 14 kDa
M
HEK293
CaSki
Vector
ISG15 OE
FGF1 16 kDa
N
CHX (h)
Flag-ISG15
O
vector
Myc-FGF1
HA-ubiquitin
DMSO
3-MA
MG132
Myc 16 kDa
P
Q
R
S
IP
IgG
Ubiquitin
shNC
shISG15
Flag 14 kDa
IP:Ubiquitin
IB:Myc 16 kDa
Input
V
shPTGDS
PTGDS 28kDa
FGF1 16,17,18 kDa
p-FGFR1 Y654 117 kDa
FGFR1 92,120,145 kDa
p-PI3K p85 Y458/p55 Y199 60,85 kDa
PI3K p85 85 kDa
p-AKT S473 60 kDa
p-AKT T308 60 kDa
AKT 60 kDa
p-mTOR S2448 288 kDa
mTOR 288 kDa

Figure S5

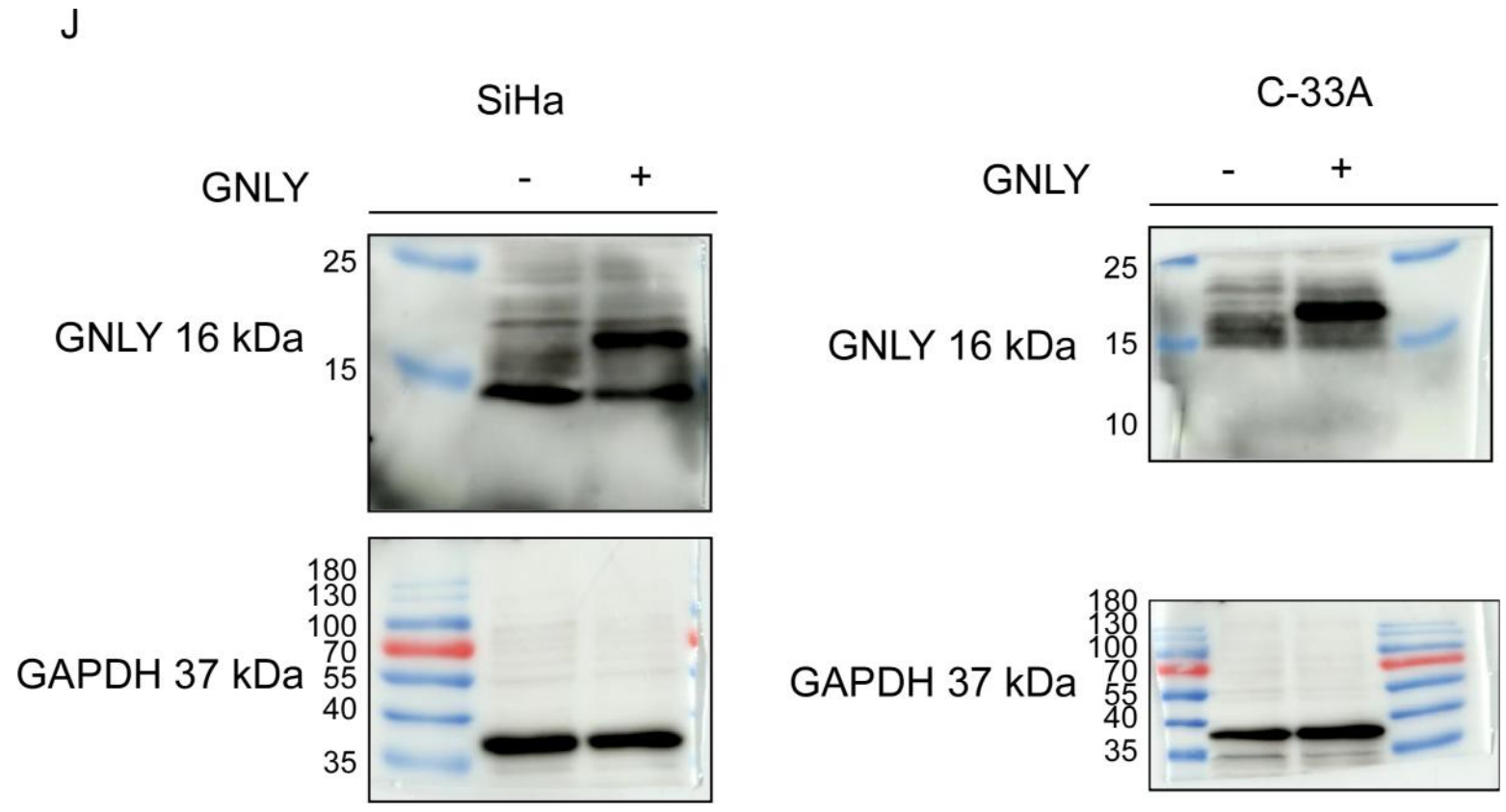

Figure S12

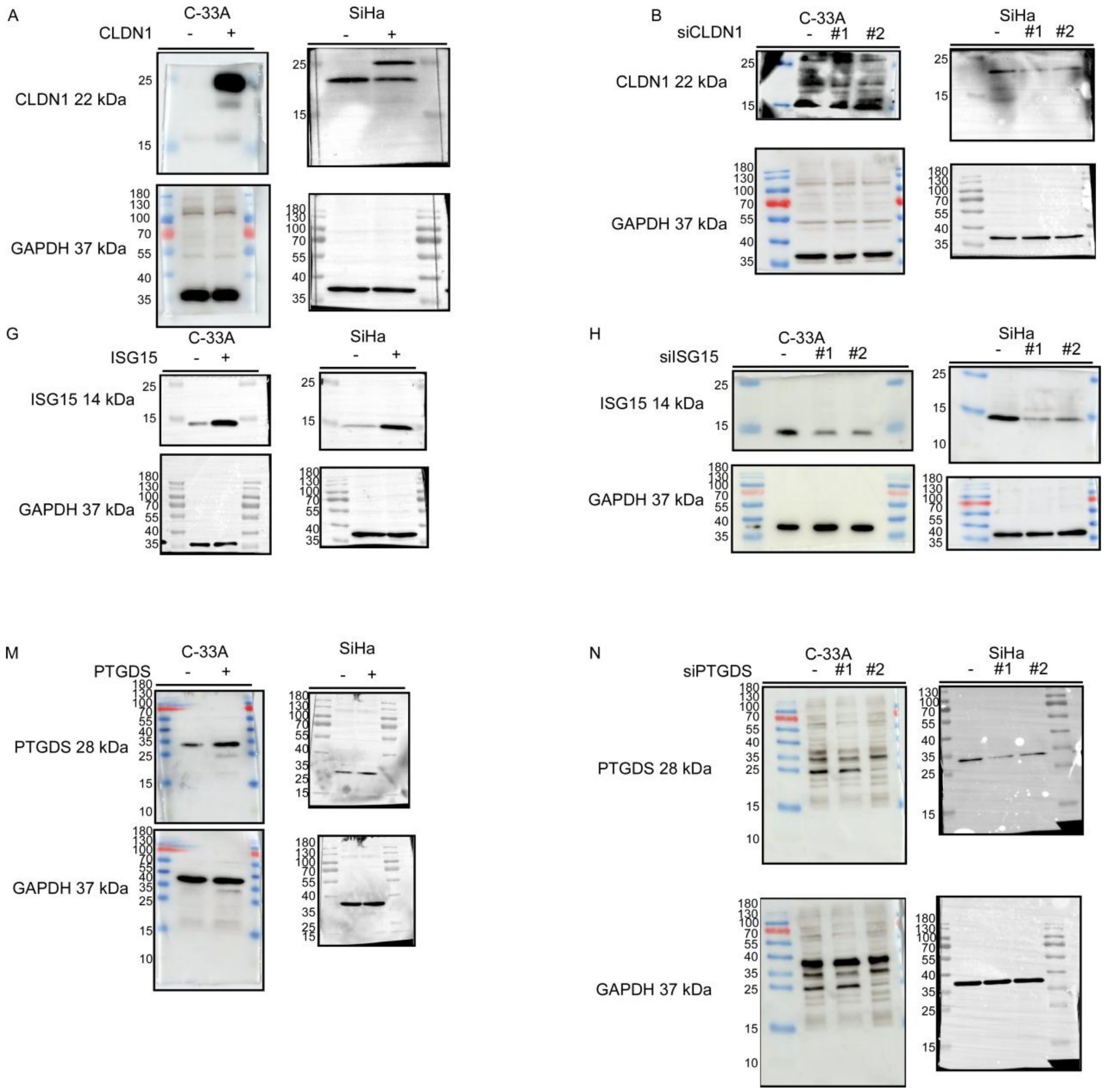

Supplement: Supplementary file 1 [file 41419_2026_8960_MOESM2_ESM.pdf]
