# Supplementary material for: Integrated multi-omics profiling of treatment-naive cervix uteri premalignant lesions and cervical squamous cell carcinoma reveals ecosystem and drivers underlying cervical cancer progression
Source: Cell Death Dis. 2026 Jun 9;17(1):722. doi: 10.1038/s41419-026-08960-2 (PMC13469616; doi:10.1038/s41419-026-08960-2)
Supplement: Supplementary file 2 [file 41419_2026_8960_MOESM1_ESM.docx]

**Supplementary materials**

Figure S1. Seurat clusters of normal cervical tissue, cervical premalignant lesions, and malignant lesions.

A-D. CellRanger t-SNE clusters of single samples, NCU (A), HGCIN (B), CCIS (C), and CSCC (D).

E. CellRanger t-SNE clusters of integrated samples.

F. CellRanger t-SNE plot of integrated samples.

G. CellRanger t-SNE clusters of the integrated samples, separately.

H. Statistical diagram of cell clusters distribution in different samples.

I. Bubble map of top two differentially expressed genes in each cluster. The darker the color, the higher the average expression value of the gene, the larger the dot and the larger the expression ratio. Because of possible overlap in the top two differentially expressed genes between clusters, the actual number of unique genes may be lower.

Figure S2. Cell communication of premalignant and malignant lesions of cervix uteri.

A-D. Heatmaps of intercellular receptor-ligand interactions showing the number of related interactions in different cell types (A, NCU; B, HGCIN; C, CCIS; D, CSCC). The rows and columns represent cell types, and each grid cell represents the number of interactions between cells. The redder the color, the greater the interactions between cells, indicating a stronger intercellular communication.

E. Intercellular communication network of integrated samples.

F. Heatmaps of intercellular ligand-receptor interactions in the integrated samples.

Figure S3. Integrated single-cell atlas of normal, premalignant, and malignant lesions of cervix uteri.

A. t-SNE plot of cells to visualize cell-type clusters based on the expression of known marker genes in a single sample.
B. t-SNE plot of cells to visualize cell-type clusters based on the expression of known marker genes in the integrated samples.
C. Statistical chart of cell type distribution in different samples.
D. Heatmap of cell-type markers. The relative expression level of genes across cells is presented, sorted by cell type. Cell-type marker genes were identified in an unbiased manner (Wilcoxon rank-sum test, thred < 0.1, FDR as parameter, and fold change ≥ 2).

Figure S4. Combined analysis of spatial transcriptomics and single-cell RNA sequencing data.

A. Scatter plot of scRNA-seq and ST expression data in different samples. The horizontal axis represents log_10_ of gene expression value of each spot, and the vertical axis represents log_10_ of gene expression value of each cell.
B. Heatmap shows the enrichment of cell types in each tissue region (score). Subgroups of functional cells are listed as spatial organization region, each unit value is *p-*value or score, score = -log_10_^(^*^p-^*^value+ 1E-100)^.
C. Heatmap shows the enrichment of cell types in each tissue region (*p*-value).

Figure S5. scRNA-seq and spatial transcriptome profiles of fibroblast subtypes.

A. Volcano plot of feature genes in vascular cancer associated fibroblasts (vascular CAFs).
B. Enriched biological processes of feature genes in vascular CAFs.
C. Enriched KEGG pathways of feature genes in vascular CAFs.
D. Expression of vascular CAFs marker genes TAGLN and MYH11 in normal and cervical cancer tissues.
E. Volcano plot of feature genes in immune CAFs.
F. Biological processes of feature genes in immune CAFs.
G. KEGG pathways of feature genes in immune CAFs.
H. Expression of immune CAFs marker genes GNLY and GZMA in normal and cervical cancer tissues.
I. GNLY expression profiling was identified in GEO database (GSE7803). The samples included 10 normal squamous cervical epithelial samples and 21 invasive squamous cell carcinoma samples of the cervix.
J. GNLY overexpression in multiple cervical cancer cells.

K. Proliferation of GNLY overexpressed cells was assessed using MTT assay.

L, M. Colony formation of GNLY overexpressed cells was verified by crystal violet staining. Statistical significance was determined by Student’s t-test. The *p*-value < 0.05 was considered statistically significant (*p* < 0.05: *, *p* < 0.01: **, *p* < 0.001: ***, *p* < 0.0001: ****).

Figure S6. scRNA-seq and spatial transcriptome profiles of endothelial cell subtypes.

A. Volcano plot of feature genes in extracellular matrix endothelial cells (ECM-ECs).
B. Enriched biological processes of feature genes in ECM-ECs.
C. Enriched KEGG pathways of feature genes in ECM-ECs.
D. Expression of ECM-ECs marker genes SELE and ACKR1 in normal and cervical cancer tissues.
E. Volcano plot of feature genes in immune ECs.
F. Biological processes of feature genes in immune ECs.
G. KEGG pathways of feature genes in immune ECs.
H. Expression of immune ECs marker genes GNLY and GZMA in normal and cervical cancer tissues.
I. The RNA velocity analysis plot shows the trajectory of CAF subpopulations, with Cluster 3 (Vascular CAFs, blue) and Cluster 4 (Immune response CAFs, red). The arrows indicate the predicted direction of cell state transitions based on RNA velocity.
J. RNA velocity analysis reveals the trajectory of EC subpopulations, with Cluster 0 (ECM-ECs, red) and Cluster 6 (Immune ECs, blue). Arrows indicate the predicted direction of cell state transitions.

Figure S7. GNLY-COL3A1 fibroblasts and GNLY-VWF endothelial cells contribute to the precancer-to-cancer transition in CSCC.

A. Laser confocal images of GNLY and COL3A1 in the tissues from the same patients diagnosed with HGCIN previously and CSCC ultimately. Scale bars: 50 μm.

B. Confocal laser microscopy images showing GNLY and VWF in tissues from patients who were initially diagnosed with HGCIN and later developed CSCC. Scale bars: 50 μm.

Figure S8. scRNA-seq and spatial transcriptome profiles of B-cell subtypes.

A. Volcano plot of feature genes in basal-like B cells.
B. Enriched biological processes of feature genes in basal-like B cells.
C. Enriched KEGG pathways of feature genes in basal-like B cells.
D. Expression of basal-like B cell marker genes S100A9 and KRT14 in normal and cervical cancer tissues.
E. Volcano plot of feature genes in progenitor cells.
F. Biological processes of feature genes in progenitor cells.
G. KEGG pathways of feature genes in progenitor cells.
H. Expression of progenitor cell marker genes IGFBP7 and C11orf96 in normal and cervical cancer tissues.
I. RNA velocity analysis reveals the dynamics of B cell subpopulations, with Cluster 4 (red, Basal-like B cells) and Cluster 5 (Progenitor cells, blue). Arrows indicate the predicted direction of cell state transitions based on RNA velocity.

J. Laser confocal images of IGFBP7 and IGKC in the tissues from the same patients, initially diagnosed with HGCIN and later with CSCC. Scale bars: 50 μm.

Figure S9. PTGDS, CLDN1, ISG15 expression are dysregulated in PCT of CSCC.

A. Heatmap of dysregulated genes analyzed from scRNA-seq data (70 genes are presented).

B. Heatmap of dysregulated genes enriched from spatial transcriptomics data (70 genes are shown).

C. Spatial distribution and expression of IGKC, CLDN1, PTGDS, ISG15, CDKN2A, and MKI67 in different tissues.

D. Violin plots of IGKC, CLDN1, PTGDS, ISG15, CDKN2A, and MKI67 expression levels in different tissues.

Figure S10. Expression of PTGDS, CLDN1, ISG15, and IGKC in cervical cancer and normal cervical tissues.

A-D. Expression of CLDN1, PTGDS, ISG15, and IGKC in different cancer types in the GEPIA database. (red: significantly upregulated, green: significantly downregulated)

E. Expression of CLDN1, PTGDS, and ISG15 in TCGA database.

F. Percentage survival of cervical cancer patients with low and high PTGDS expression from GEPIA database.

G. Correlation between the expression of CLDN1, PTGDS, ISG15, IGKC and clinical features of cervical cancer patients, based on data from the MEXPRESS database. Statistical significance was determined by one-tailed Student’s t-test. The *p* < 0.05 was considered statistically significant (*p* < 0.01: **, *p* < 0.001: ***).

Figure S11. Expression of CLDN1, PTGDS, and ISG15 is associated with precancer-to-cancer transition in CSCC.

A. Relative mRNA expression levels of CLDN1, PTGDS, ISG15, IGKC, and SCCA in the serum of normal, precancer, and cancer patients.
B. mIHC staining images of CLDN1/ISG15 in precancer and cancer tissues from the same patients diagnosed at different time points. Scale bars: 50 µm.
C. mIHC staining images showing CLDN1/PTGDS in precancer and cancer tissues of the same patients at different stages of diagnosis. Scale bars: 50 µm. Statistical significance was determined by one-way ANOVA (*p* < 0.05: *, *p* < 0.01: **).

Figure S12. Function of CLDN1, ISG15, and PTGDS in cervical cancer cells.

A, B. Overexpression and knockdown of CLDN1 in different cervical cancer cells.

C, D. Proliferation of CLDN1 overexpressed and downregulated cells was assessed by MTT assay.

E, F. Colony formation of CLDN1 overexpressed and downregulated cells was evaluated by crystal violet staining.

G, H. ISG15 expression after overexpressing and knocking down was identified by Western blotting.

I, J. Proliferation of ISG15-overexpressed and ISG15-knockdown cell was assessed by MTT assay.

K, L. Colony formation of ISG15-overexpressed and ISG15-knockdown cells.

M, N. Expression of PTGDS in overexpressed and knockdown cells.

O, P. Cell proliferation of PTGDS upregulated and downregulated cells.

Q, R. Colony formation of PTGDS upregulated and downregulated cells.

Statistical significance was determined by Student’s t-test (C, E, I, K, O, Q) and one-way ANOVA (D, F, J, L, P, R). The *p* < 0.05 was considered statistically significant (*p* < 0.05: *, *p* < 0.01: **, *p* < 0.001: ***, *p* < 0.0001: ****).

Figure S13. PTGDS, CLDN1, and ISG15 regulate the FGF1/FGFR1/PI3K/AKT signaling pathway in cervical cancer.

A. Venn diagram of transcriptomic sequencing data using PTGDS-, CLDN1-, and ISG15-overexpressed cells.

B. Signaling pathways regulated by PTGDS, CLDN1, and ISG15 as identified through transcriptomic enrichment analysis.

C-E. KEGG pathway enrichment based on scRNA-seq data in endothelial cells (C), fibroblasts (D), and B cells (E).

F-J. Expression of PI3K/AKT signaling pathway related markers (PIK3CA, F; AKT1, G; AKT2, H; AKT3, I; MTOR, J) in different samples.

Figure S14. ISG15 expression in different CAFs during PCT.

A. Volin plots of ISG15 expression in CAFs subsets in NCU, HGCIN, CCIS, and CSCC tissues.

B. Violin plot of ISG15 expression in inflammatory CAFs in NCU, HGCIN, CCIS, and CSCC tissues.

C. Diagram of the intercellular receptor-ligand interaction network in CSCC tissues shows the number of ligand-receptor interactions detected between malignant epithelial cells and fibroblast subclusters.

D. Images of colony formation assays in cervical cancer cells treated with various concentrations of PD161570.

Figure S15. CLDN1 expression is associated with poor prognosis in cervical cancer patients.

A. Immunohistochemical staining of CLDN1 in cervical cancer tumor tissues and adjacent normal tissues with cervical cancer tissue array (HUteS120Su01). Representative images are shown at 40× and 100× magnification. Scale bars: 200 µm and 50 µm.

B. CLDN1 expression in cervical cancer tumor tissues and adjacent normal tissues was evaluated using IHC staining with cervical cancer tissue array.

C. CLDN1 expression in paired cervical cancer tumor tissues and adjacent normal tissues was analyzed by tissue array (HUteS30PG01-2).

D. Overall survival of cervical cancer patients with different CLDN1 expression levels was analyzed by tissue array (HUteS120Su01). Statistical significance was determined by Student’s t-test. The *p*-value < 0.05 was considered statistically significant. Levels of significance were denoted as follows: *p* < 0.05: *, *p* < 0.01: **, *p* < 0.001: ***, *p* < 0.0001: ****.

Figure S16. CLDN1, ISG15, PTGDS regulate cervical cancer tumor growth via the FGFR1/PI3K/AKT/mTOR pathway.

A. Representative IHC images of CLDN1, Ki67, p-FGFR1 (Y654), p-AKT (S473), p-mTOR (S2448) in CLDN1-overexpressed CDX tumor tissues derived from C-33A cells. Scale bars: 100 µm.

B. Representative IHC images showing the expression of ISG15, Ki67, p-FGFR1 (Y654), p-AKT (S473), p-mTOR (S2448) in ISG15-overexpressed CDX tumor tissues derived from C-33A cells. Scale bars: 100 µm.

C. Representative IHC images showing PTGDS, Ki67, p-FGFR1 (Y654), p-AKT (S473), and p-mTOR (S2448) in CDX tumor tissues derived from C-33A cells with PTGDS overexpression. Scale bars: 100 µm.

D. Quantification of CLDN1, Ki67, p-FGFR1 (Y654), p-AKT (S473), and p-mTOR (S2448) in CLDN1-overexpressed CDX tumor tissues derived from C-33A cells by IHC.

E. Quantitative analysis of ISG15, Ki67, p-FGFR1 (Y654), p-AKT (S473), and p-mTOR (S2448) expression in ISG15-overexpressed CDX tumor tissues by IHC.

F. PTGDS, Ki67, p-FGFR1 (Y654), p-AKT (S473), and p-mTOR (S2448) levels were evaluated in PTGDS-overexpressed CDX tumors assessed by IHC, with corresponding statistical quantification shown. Statistical significance was determined by Student’s t-test. The *p*-value < 0.05 was considered statistically significant. Significance levels were denoted as follows: *p* < 0.05: *, *p* < 0.01: **, *p* < 0.001: ***, *p* < 0.0001: ****.

Figure S17. PD161570 inhibits cervical cancer tumor growth via FGFR1/PI3K/AKT/mTOR pathway.

A, B. Immunohistochemical images and quantification of Ki67 expression in CDX tumors derived from C-33A cells. Mice in different groups were treated with PBS, alpelisib (40 mg/kg), everolimus (1 mg/kg), and PD161570 (2 mg/kg). Scale bars: 50 µm.

C, D. IHC images and statistical analysis of p-FGFR1 (Y654), p-AKT (S473), and p-mTOR (S2448) expression in CDX tumors derived from C-33A cells. Mice in different groups were treated with PBS and PD161570 (2 mg/kg). Scale bars: 50 µm.

E. H&E staining of the heart, liver, spleen, lung, and kidney in CDX mice models. Mice were treated with PBS, alpelisib (40 mg/kg), everolimus (1 mg/kg), and PD161570 (2 mg/kg). Scale bars: 100 µm.

F-J. Boxplots of CLDN1 expression comparing responder and non-responder samples in a combined dataset of anti-PD-1 (F), Nivolumab (G), Pembrolizumab (H), anti-PD-L1 (I), or any immune checkpoint inhibitor therapy (ANY) (J) pretreatment samples. The upper annotations indicate the *p*-value, AUC and strongest cutoff value based on ROC analysis in binary classification for predicting sensitivity to immunotherapy.

K-M. Boxplots of ISG15 expression comparing responder and non-responder samples in a combined dataset of anti-PD-1 (K), anti-CTLA-4 (L), Ipilimumab (M) pretreatment samples. The upper annotations indicate the *p*-value, AUC and strongest cutoff value based on ROC analysis in binary classification for predicting sensitivity to immunotherapy. Statistical significance was determined by one-way ANOVA (B) and Student’s t-test (D). The *p*-value < 0.05 was considered statistically significant. Significance levels were defined as follows: *p* < 0.05: *, *p* < 0.01: **, *p* < 0.001: ***, *p* < 0.0001: ****.
